# Structural Forces in Ionic Liquids: The Role of Ionic Size Asymmetry

**DOI:** 10.1021/acs.jpcb.1c09441

**Published:** 2022-02-08

**Authors:** J. Pedro de Souza, Karina Pivnic, Martin Z. Bazant, Michael Urbakh, Alexei A. Kornyshev

**Affiliations:** † Department of Chemical Engineering, Massachusetts Institute of Technology, Cambridge, Massachusetts 02139, United States; ‡ School of Chemistry, The Sackler Center for Computational Molecular and Materials Science, Tel Aviv University, Tel Aviv, 6997801, Israel; ¶ Department of Mathematics, Massachusetts Institute of Technology, Cambridge, Massachusetts 02139, United States; § Department of Chemistry, Molecular Sciences Research Hub, Imperial College London, London, W12 0BZ 2AZ, United Kingdom; ∥ Thomas Young Centre for Theory and Simulation of Materials, Imperial College London, South Kensington Campus, London, SW7 2AZ, United Kingdom

## Abstract

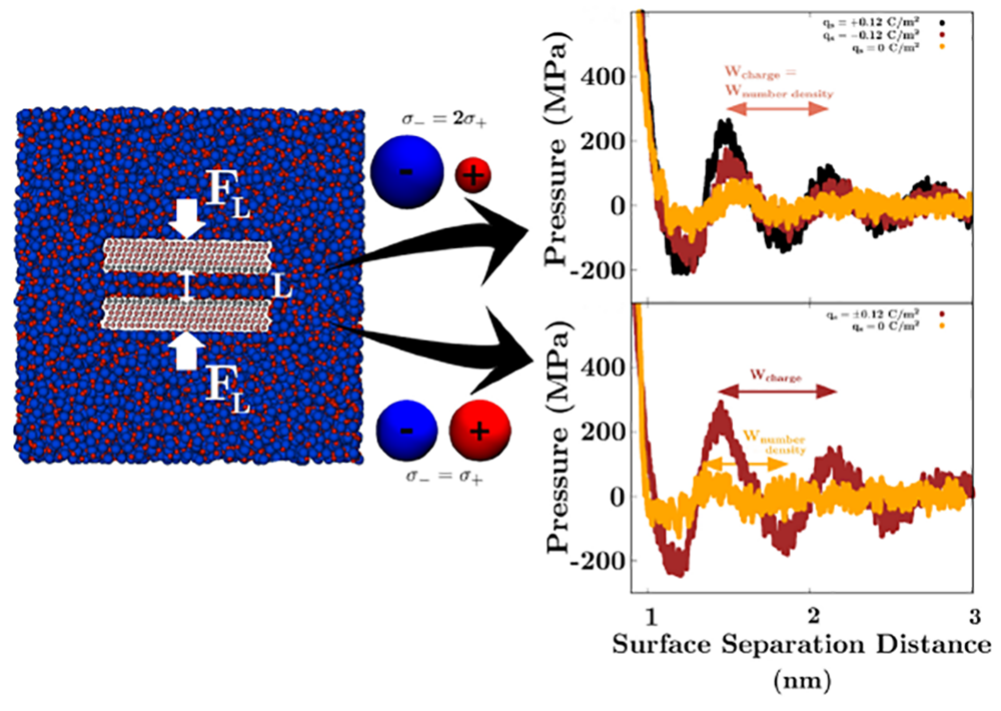

Ionic liquids (ILs) are charged fluids composed of anions and cations of different size and shape. The ordering of charge and density in ILs confined between charged interfaces underlies numerous applications of IL electrolytes. Here, we analyze the screening behavior and the resulting structural forces of a representative IL confined between two charge-varied plates. Using both molecular dynamics simulations and a continuum theory, we contrast the screening features of a more-realistic asymmetric system and a less-realistic symmetric one. The ionic size asymmetry plays a nontrivial role in charge screening, affecting both the ionic density profiles and the disjoining pressure distance dependence. Ionic systems with size asymmetry are stronger coupled systems, and this manifests itself both in their response to the electrode polarization and spontaneous structure formation at the interface. Analytical expressions for decay lengths of the disjoining pressure are obtained in agreement with the pressure profiles computed from molecular dynamics simulations.

## 1 Introduction

Room-temperature ionic liquids (ILs) are molten salts composed of majorly asymmetrically sized anions and cations.^1,2^ ILs have wide electrochemical stability windows, low vapor pressure, and are thermally stable.^3,4^ Because of their exceptional properties, they are used in energy storage applications including supercapacitors and batteries^3^ and as solvents for reactions and for catalysis.^2^ They can also be employed as electrotunable lubricants.^5,6^ In these applications, the ILs can be confined in charged nanopores down to the nanometer scale, in which the extent of the nanopore confinement and its polarity determine the interfacial IL structure and charge layering.^7−10^

To optimize the interfacial behavior of ILs for their many applications, researchers need to accurately model ILs. This is done either through computationally expensive atomistic simulations or via sophisticated theoretical approaches, which go beyond standard mean-field theories of dilute electrolyte solutions. In such highly concentrated electrolytes as ILs, the dilute solution theory is predestined to fail since ILs form structures determined by dense packing of ions in the *crowding*(11−16) (layering of ions of the same charge at highly charged electrodes) and *overscreening*(17,18) (alternating layers of opposite charge at weakly charged electrodes) regimes. Most egregiously, the dilute solution theoretical description does not take into account the ionic size.^11,19,20^ To capture these screening features, multiple continuum theories have been developed to include the finite size of ions in their steric and electrostatic interactions, especially for concentrated systems at high voltages.^21−26^ The simplest theoretical models of these systems have been developed for the case of ions of the same size. This was natural to do as a start, although most ILs exhibit strong size asymmetry. In theories, ion asymmetry has typically been studied within the mean field approach that accounted for crowding, particularly in the explanation of the asymmetry of the double-layer differential capacitance curves in such systems.^3,12,27−31^ In molecular dynamics (MD) simulations, ion asymmetry has been either specially introduced^18^ or naturally included with fully atomistic or coarse grained models of ions.^3^ Certain classical Density Functional Theories (DFT) have also been applied to asymmetric ILs, predicting interfacial layering in line with MD simulations.^24,32^ These studies of ILs draw from the wide body of theoretical research on primitive model electrolytes either through classical DFT^33−37^ or integral equation theories.^38−43^ In fact, earlier work from Greberg and Kjellander revealed the role of asymmetry in the contact behavior and decay of bulk correlations in primitive model electrolytes.^44^

Surface force apparatus (SFA) and atomic force microscopy (AFM) measurements have emerged as the main experimental tools to investigate the nanoscale structure of interfacial liquids,^45^ including ILs.^7,8,46,47^ While in SFA experiments the mica surfaces are spontaneously negatively charged, the AFM setup can incorporate conductive electrodes, allowing for the independent control of charges on the surfaces. Both SFA and AFM measurements have been performed in a variety of ILs, and in all cases decaying oscillatory forces were observed, with an oscillation period of the order of an ion pair diameter (usually dominated by the largest ion diameter), indicative of the underlying alternating charge layering structure.^8,9,47,48^ At even longer ranges than the oscillatory forces, SFA measurements found an additional monotonically decaying “tail” of the force, both in concentrated electrolytes and ILs.^48−51^

Despite these findings, however, simulations and theoretical descriptions of primitive model electrolytes have not succeeded to recover the existence of such a tail.^34^ Long-range electrostatic forces in the dilute limit can be described by analytical formulas derived from Debye–Huckel linearization, but such simple analytical equations are not readily available for the oscillatory structural forces. While the above-mentioned theories presented sophisticated analyses of the role of asymmetry on interfacial ionic behavior, they still did not present simplified, explicit formulas for the oscillatory IL structure and structural forces, nor did they directly validate the role of asymmetry on the structural forces and screening structure by MD simulations of the concentrated IL limit in varying extents of confinement. Instead of directly applying theoretically derived formulas, the experimental oscillatory forces are typically empirically fitted to an oscillatory decaying function. Therefore, the physical and quantitative interpretation of oscillatory structural forces in experiments could greatly benefit from analytical formulas derived within theories of concentrated, and generally asymmetric ILs.

In the present work, we go further into investigating the double layer structure of an asymmetric IL under confinement between equally charged surfaces using both MD simulations and an advanced continuum theory. The ion density profiles and disjoining pressure curves that we calculate based on the original theory^22^ show qualitative and quantitative agreement with the results of simulations for a range of surface charge densities and surface separations. By a comparison of a representative IL with asymmetric ions to an IL composed of size-symmetric ions, we demonstrate that the size asymmetry strongly determines the ionic layering structure between two flat charged interfaces. Even at *zero charge of the electrode* there is an entropy-driven “preferential adsorption” of smaller ions, which results in *spontaneous layering of positive and negative charges near the electrode*. The order of layering may, in fact, be changed by specific adsorption of any of these ions, which is not included in our simple model, but which could easily be modeled by adding a specific term in the interaction potential between the ions and the surface. In whatever direction that effect could have shifted the result, it would act at the background of the noted effect.

Furthermore, the size asymmetry leads to a strong coupling of charge density and number density oscillations even far from the interface that is absent for the symmetric case. Analytical approximations are shown to reproduce the simulated modes of decaying oscillations in the disjoining pressure at the nanometer scale. However, neither the simulations nor the theory contain any evidence of an abnormally long decay length or long-range exponential tails. Nevertheless, the main value of the theory lies in the analytical description of the decaying modes of the structural forces and their dependence on surface charging in terms of the IL physical properties. The numerical predictions of the full nonlinear theory can be generated with much smaller computational cost compared to atomistic simulations.

All in all, the presented theory, backed up by the MD simulations, demonstrates that the ion asymmetry in electrostatic and steric interactions are essential in describing the double layer structure for ultraconcentrated, asymmetric ILs. These findings therefore signify an important step in the advancement of our understanding of the screening behavior and the resulting structural forces of ultraconcentrated, asymmetric ILs as well as of solvent-in-salt systems, under nanoconfinements.

## 2 Simulations and Theory

In this paper, an IL is approximated in both MD simulations and a continuum theory, first with asymmetric anion and cation sizes, and then with an equal anion and cation size, as charged Lennard-Jones (LJ) spheres. Such level of simplification has been chosen first of all to investigate the effects not obscured by any chemical complexity of the ions, and secondly because this would allow the most straightforward comparison between the simulations and the theory that we use here. We highlight the properties and parameters of the MD simulations and the continuum theory before applying them to model the IL and the resulting disjoining pressures. For convenience, the symbols which appear in the theory are summarized and listed in the Glossary of Symbols at the end of the article.

### 2.1 Simulation Details

**Figure 1:**
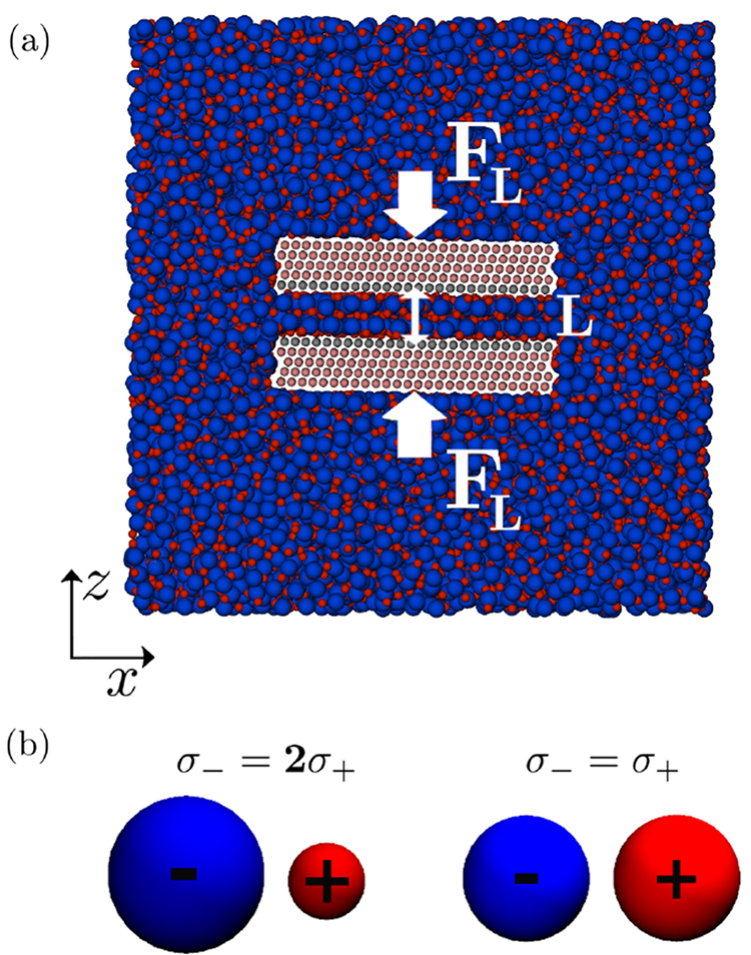
MD simulations. (a) Snapshot of the asymmetric IL immersing two charged surfaces in a fully periodic simulations box. The surfaces are pushed together along the *z*-direction with a normal force *F*_*L*_ to ultimately calculate the pressure as a function of the separation distance, *L*. (b) The ionic sizes, characterized by the LJ diameter, σ_*i*_, for the (left) asymmetric and (right) symmetric systems.

Within this minimal model, the ions are represented as a 1:1 mixture of oppositely and singly charged LJ spheres,^18,52,53^ as shown in Figure 1. The concentration of both anions and cations in the simulation box is *c*_0_ = 4.586 M and the absolute temperature is *T* = 600 K (usually elevated temperature is taken when “experimenting” with charged LJ-spheres, because at room temperature the dense plasma of such spheres freezes out). In our model, we account for the electronic polarizability of the ions in an effective manner. For this, we have set the background permittivity to be ϵ = 2ϵ_0_,^52,53^ where ϵ_0_ is the permittivity of free space, with each ion possessing one elementary charge. The ions interact through LJ and Coulombic potentials, where the size asymmetry of the ions is controlled by adjusting their diameters through the LJ parameter σ_*ij*_ for species *i* interacting with species *j*, or σ_*i*_ when *i* = *j*. The ion sizes that we consider here for the asymmetric system are σ_–_ = 0.7 nm and σ_+_ = 0.35 nm, and the ion size for the symmetric system is σ_–_ = σ_+_ = 0.58 nm, such that the filling fraction is approximately equal in both systems, making ∑_*i*_σ_*i*_^3^*c*_0_ unchanged. We also include attractive dispersion interactions. This is done in order to capture a more realistic representation of the IL, as these attractive dispersion interactions are active in real ILs. In our simulations, the cutoff distance of the LJ potential is set to be as long as 1.8 nm. Even so, as reaffirmed in the theoretical predictions which do not incorporate dispersion interactions, the main balance guiding the structural forces is the interplay between the ionic charge and ionic finite size.

Two parallel plates in the *x*–*y* plane are immersed in a bulk of IL. We consider flat surfaces comprising LJ spheres in contact with the confined liquid, and a lattice parameter of mica.^52^ Performing constant charge simulations, the surface charge on each plate is varied between *q*_*s*_ = −0.12 C/m^2^ and *q*_*s*_ = +0.12 C/m^2^. In the simulations, the image charge interactions are not taken into account. Simulations of IL nanofilms (1–10 nm thick) showed that the effect of electrode polarizability (image charges) on the vertical and lateral structure of the confined liquids is insignificant at practically feasible applied voltages.^54−56^ Image charge interactions can lead, in principle, to the depletion of ions in the nanogap for a single layer of ions confined between two uncharged or slightly charged dielectric walls, as the ions, instead of “seeing” their counterions nearby, see their own weak images in the side walls (the situation may be different for metallic surfaces^57^). However, for larger separation distances between the surfaces, there is a weak dependence of the IL’s structuring on the electrode polarizability which is explained by the screening of image charge interactions by the first layer of ions.^58^ Therefore, in our work, we choose not to include image charge interactions, as we are particularly interested in isolating the structural layering of the liquid, which occurs even without the complexity that is added with the introduction of image charge interactions.

In the simulations, for each fixed surface charge the ionic density profiles between the surfaces are computed at a fixed separation distance, and the separation distance is varied to generate a pressure curve. Expansion on the simulation details and the used methods can be found further in the Supporting Information (SI).

### 2.2 Theoretical Derivation

The theory is based on an approximation of the Helmholtz free energy of a system of an asymmetric, hard-sphere 1:1 electrolyte with a constant background permittivity, ϵ, the primitive model.^34,59−61^ The phenomenological basis for the theory is that the electrostatic and hard sphere components of the free energy of the system can be expressed in terms of locally homogenized quantities of the ion densities, the weighted-density approximation (WDA). The distinguishing feature of this model, as opposed to other similar classical DFT approaches,^62^ is that the electrostatic contribution to the free energy is directly expressed in terms of weighted ionic densities.^22,63,64^ The model is a generalization of the density functional for ILs presented in ref (22).

The core ingredients of the theory that allow us to capture the discrete layering in ILs are (i) representing the ions as delocalized shells of charge in their electrostatics and (ii) encoding the hard sphere packing of ions in their excess chemical potential. While these effects introduce some mathematical complexity, they have straightforward physical interpretations and do allow for some analytical progress, especially in the linear response limit.

To start, the free energy of the system can be broken down into three contributions, an ideal part , an excess part , and an electrostatic part :
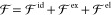
1The ideal contribution is related to the entropy of an ideal gas:
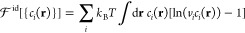
2where *k*_B_*T* is the thermal energy, *v*_*i*_ is the volume of ion *i*, and *c*_*i*_ is the number density of ion *i*. For the hard sphere contribution to the free energy, we incorporate a phenomenological, simplified version of the Fundamental Measure Theory (FMT).^65^ The advantage of our approach is that more compact expressions can be derived for the ionic excess chemical potential in terms of fewer weighting functions, aiding in the process of deriving simplified analytical approximations to the theory.

The phenomenological excess free energy we define is given by
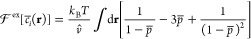
3where *p̅* is the weighted volumetric filling fraction,
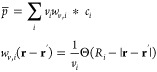
4where *R*_*i*_ is the effective hard-sphere radius of the ion, *v*_*i*_ = 4*πR*_*i*_^3^/3 is the volume of the ion, the asterisk (∗) denotes a convolution, *w*_*v*,*i*_ is the volumetric weighting function, Θ represents the Heaviside step function, and the filling-fraction-weighted volume *v̂* is given by
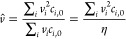
5Here, *c*_*i*,0_ is the bulk concentration of species *i* and η is the bulk filling fraction, η = ∑_*i*_*v*_*i*_*c*_*i*,0_. By construction, the key criteria that the simplified form of the hard sphere excess free energy satisfies are: (i) it retains the Carnahan–Starling equation of state^66^ for the limit of symmetric ions or where one ionic species becomes vanishingly small while the other is of finite size, and (ii) it maintains the same singularities as the FMT functional.

The electrostatic part of the free energy in a medium with dielectric constant ϵ is expressed in terms of the electrostatic potential, ϕ, and the homogenized charge density, ρ̅_*e*_:^22^
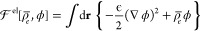
6This corresponds to the modified form of the Poisson equation, via minimization of the functional with respect to the electrostatic potential, :
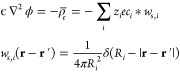
7where *z*_*i*_ is the valency of ion *i*, *e* is the elementary charge, and δ() denotes the delta function of 1D argument, such that each ionic weighting function *w*_*s*,*i*_ corresponds to homogenizing over the surface of the ionic spheres. The weighted ionic concentrations signify that the ions act as shells of charge that interact with the local electrostatic potential.

Minimizing the free energy functional with respect to concentration, the ion densities at equilibrium satisfy:
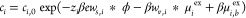
8where β is the inverse thermal energy and the excess chemical potential, μ_*i*_^ex^, is defined as
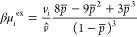
9taking on a value in the bulk, μ_*i,b*_^ex^, of
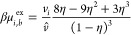
10We again note that *v*_*i*_ = *v̂* for the case where (i) the two ions have the same size or (ii) when one ion is vanishingly small while the other has finite size. These limits give the standard expression for the Carnahan–Starling equation of state. For asymmetric ILs in which the ions both have significant packing effects, the formula effectively interpolates between these two limits.

We solve the above coupled integro-differential eqs 7 and 8 for the ionic densities and electrostatic potential between two flat electrodes, with equal surface charge density, *q*_*s*_. In this case, the standard boundary condition for the potential is applied **n̂**·ϵ∇ϕ|_*s*_ = −*q*_*s*_. In the theory, the surface is assumed to be perfectly flat and hard with smeared charge density. In the theory, the representative surfaces are defined at *z* = ± *L*/2 ∓ *R*_*s*_, where *L* is the distance between surface atom centers in the simulation and *R*_*s*_ is the surface atom radius. Therefore, the theoretical ionic densities are zero for *z* < −*L*/2 + *R*_*s*_ + *R*_*i*_ and *z* > *L*/2 – *R*_*s*_ – *R*_*i*_. We solve for the area averaged density, and we therefore reduce all equations to be dependent on one coordinate, *z*.^22^ Numerically, we discretize the equations by a finite difference scheme.

For consistency between the simulations and theory, we need to define the ion diameter in the theory, *d*_*i*_, as compared to the σ_*ij*_ values of the simulation. Since the LJ interaction is not exactly the same as the hard sphere interaction assumed in the theory, the ions in the simulation can overlap slightly with the surface and with each other below a center-to-center separation of σ_*i*_. We assume that the effective ionic diameter in the theory is set by a cutoff criterion, where the LJ potential is *U*_LJ_ = 0.3*k*_B_*T*, corresponding to *d*_*i*_ ≈ 0.9σ_*i*_. The sensitivity of the results with respect to this criterion is discussed in the SI.

The pressure between the two equally charged surfaces as a function of separation distance is calculated as^67−70^
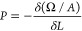
11at constant temperature and reference chemical potential (constant bulk ionic concentrations), where *A* is the area of the surfaces, and Ω is the grand potential
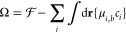
12and μ_*i,b*_ is the bulk chemical potential. By definition, the pressure corresponds to the change in the grand potential per differential change in the system volume, assuming fixed area of the confining surfaces. The values of Ω/*A* for a range of separation distances are numerically computed, and we then numerically differentiate this function to calculate the disjoining pressure. The zero value for the disjoining pressures corresponds to the bulk reference value as *L* → ∞, *P*_∞_ = 0.

## 3 Results and Discussion

### 3.1 Ionic Charge and Density Profiles

**Figure 2:**
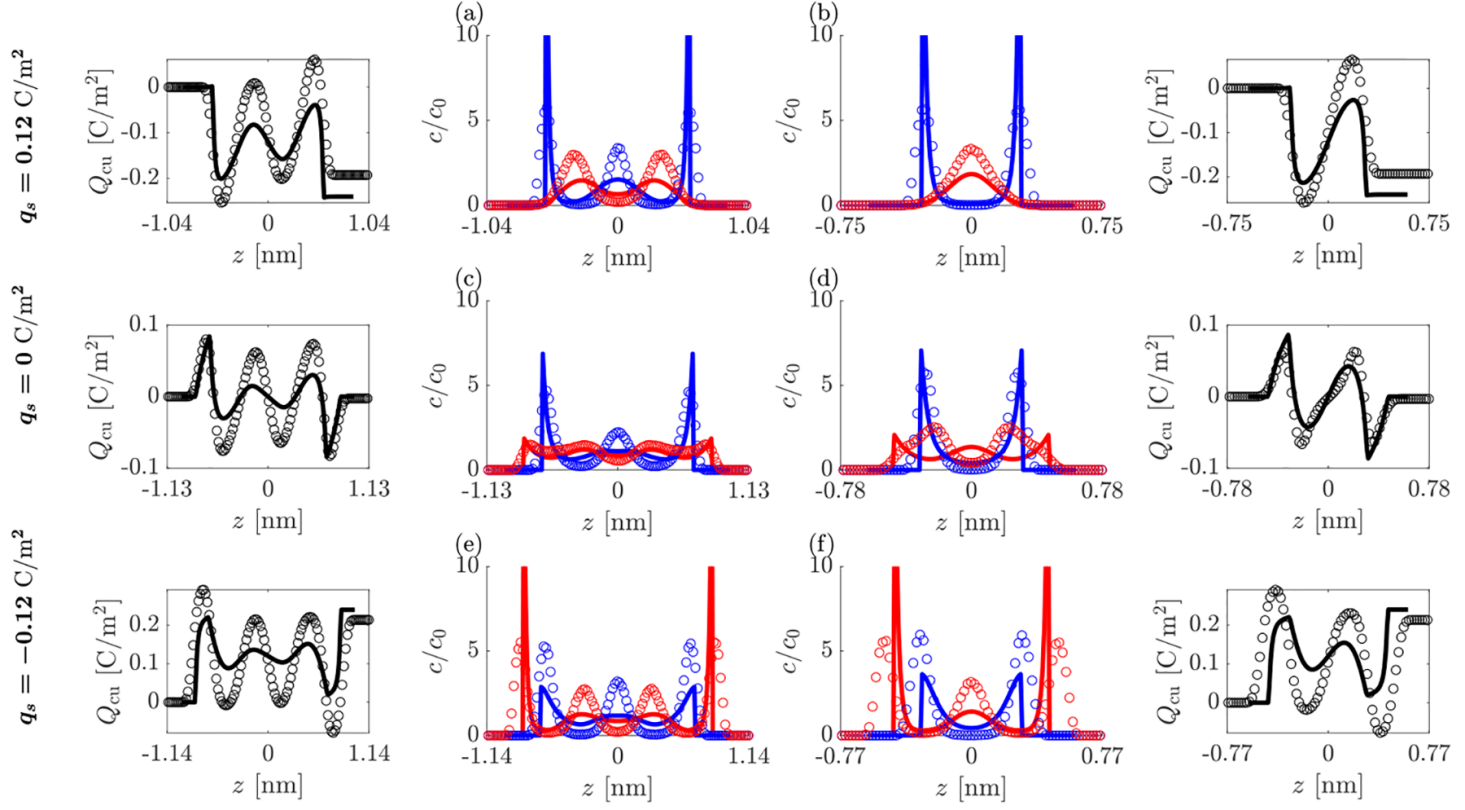
Charge and ion density profiles in asymmetric ILs between the charged plates. Rows correspond to the fixed surface charge densities of (a,b) *q*_*s*_ = 0.12 C/m^2^, (c,d) *q*_*s*_ = 0 C/m^2^, and (e,f) *q*_*s*_ = −0.12 C/m^2^. Columns correspond to the squeeze out of a central electroneutral layer between two stable states where (a,c,e) *L* ≈ 2.2 nm and (b,d,f) *L* ≈ 1.5 nm. Cumulative charge functions are plotted to the left or right of the corresponding concentration profile plot. Markers (○) simulations; lines (—) theory. Color coding: blue, anions; red, cations.

We start by comparing in Figure 2 the ionic densities calculated from the simulations (circles, ○) and theory (lines, —) as the ionic layers are squeezed out (columns, panels a–f) at varying surface charge/polarities (rows). Beyond simply plotting the density of ionic centers, *c*_*i*_, layering is demonstrated by plotting the cumulative charge (accompanying plots to the left and right of panels a–f), with the cumulative charge function, *Q*_cu_(*z*), defined as
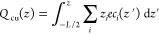
13

Figure 2 shows that the theory captures the main features of the charge ordering found in the simulations, particularly when plotted in terms of *Q*_cu_(*z*). However, the theory does not accurately predict the magnitude of the overscreening as the separation distance increases, and underpredicts the decay into the bulk for the widest separations. Interestingly, the theory is more in line with simulations when describing the charge ordering at small separations between the plates, where the bulk correlations of the ions are the least influential.

The main discrepancies between the theory and simulations are the sharpness and magnitude of the ionic density peaks, arising because the theory assumes hard-sphere interactions while the simulations assume LJ interactions. Further, whereas the theory obeys electroneutrality within the space between the two charged surfaces (*Q*_cu_(*z* = *L*/2) = −2*q*_*s*_), the simulations exhibit some partial charge screening from the IL outside the gap, so that *Q*_cu_(*z* = *L*/2) ≠ −2*q*_*s*_.

Inspecting the density profiles in Figure 2, we can see that at zero surface charge (panels c and d), both the theory and the simulations show that the smaller ion, the cation in our study (displayed in red color), can access the surface more easily. For negatively charged surfaces (panels e and f), the small cation concentration is enhanced drastically near the surfaces, yet the same number of layers are maintained as in the zero charge case. At a positive surface charge (panels a and b), the cation is pushed out of the region closest to the surfaces, resulting in fewer layers of ions in this limit.

**Figure 3:**
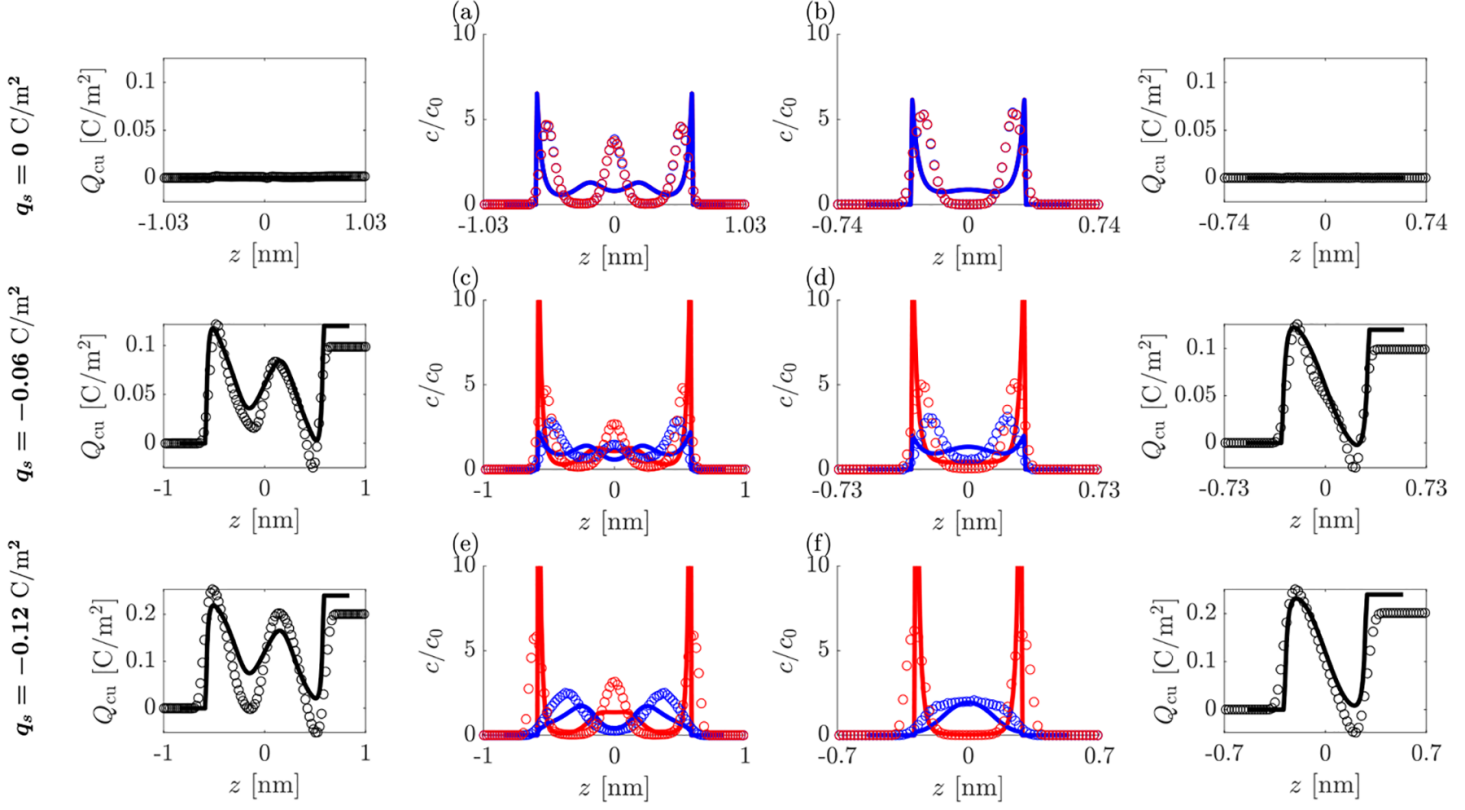
Charge and ion density profiles in symmetric IL. Rows correspond to the fixed surface charge of (a,b) *q*_*s*_ = 0 C/m^2^, (c,d) *q*_*s*_ = −0.06 C/m^2^, and (e,f) *q*_*s*_ = −0.12 C/m^2^. Columns correspond to the squeeze out of a central electroneutral layer between two stable states where (a,c,e) *L* ≈ 2 nm and (b,d,f) *L* ≈ 1.4 nm. Cumulative charge functions are plotted to the left or right of the corresponding concentration profile plot. Markers (○) simulations; lines (—) theory. Color coding: blue, anions; red, cations.

We can then contrast the screening with asymmetric ions to screening with symmetric ions. In Figure 3, the ionic density profiles for the symmetric system are shown, for increasing negative surface charge and two separation distances. Since a change in the surface polarity gives identical profiles (up to the identity of the symmetric ions) only negative surface charge values are shown. Again, overall, the theory captures ordering in charge and density between the charged surfaces quite well, with close agreement for the smallest separation distances. At zero surface charge (panels a and b), here, unlike the asymmetric ionic system, no local ionic charge density occupies the gap, since neither ion preferentially accesses the surface. The ions then form overlapping layers, which turn into alternating ones when increasing the surface charge (panels c–f). Overall, the overscreening structure remains similar as the surface charge magnitude increases, since the surface charges tested are not large enough to enter the crowding regime.

Thus, after comparing the density profiles between confined asymmetric and symmetric ionic systems we find that the most pronounced effect for the asymmetric ionic system is an entropy-driven preferential adsorption of smaller ions which emerges even at zero surface charge of the electrodes. This spontaneous layering of charges near the zero charged electrodes is completely nontrivial and it essentially emphasizes the strong effect that the ionic size asymmetry has on the layering structure of positive and negative charges near flat charged interfaces. Additional charge and density profiles with varying surface charges and separation distances for both the asymmetric and symmetric systems are presented in the SI.

### 3.2 Disjoining Pressure Profiles

#### 3.2.1 Simulations and Integro-differential Theoretical Results

**Figure 4:**
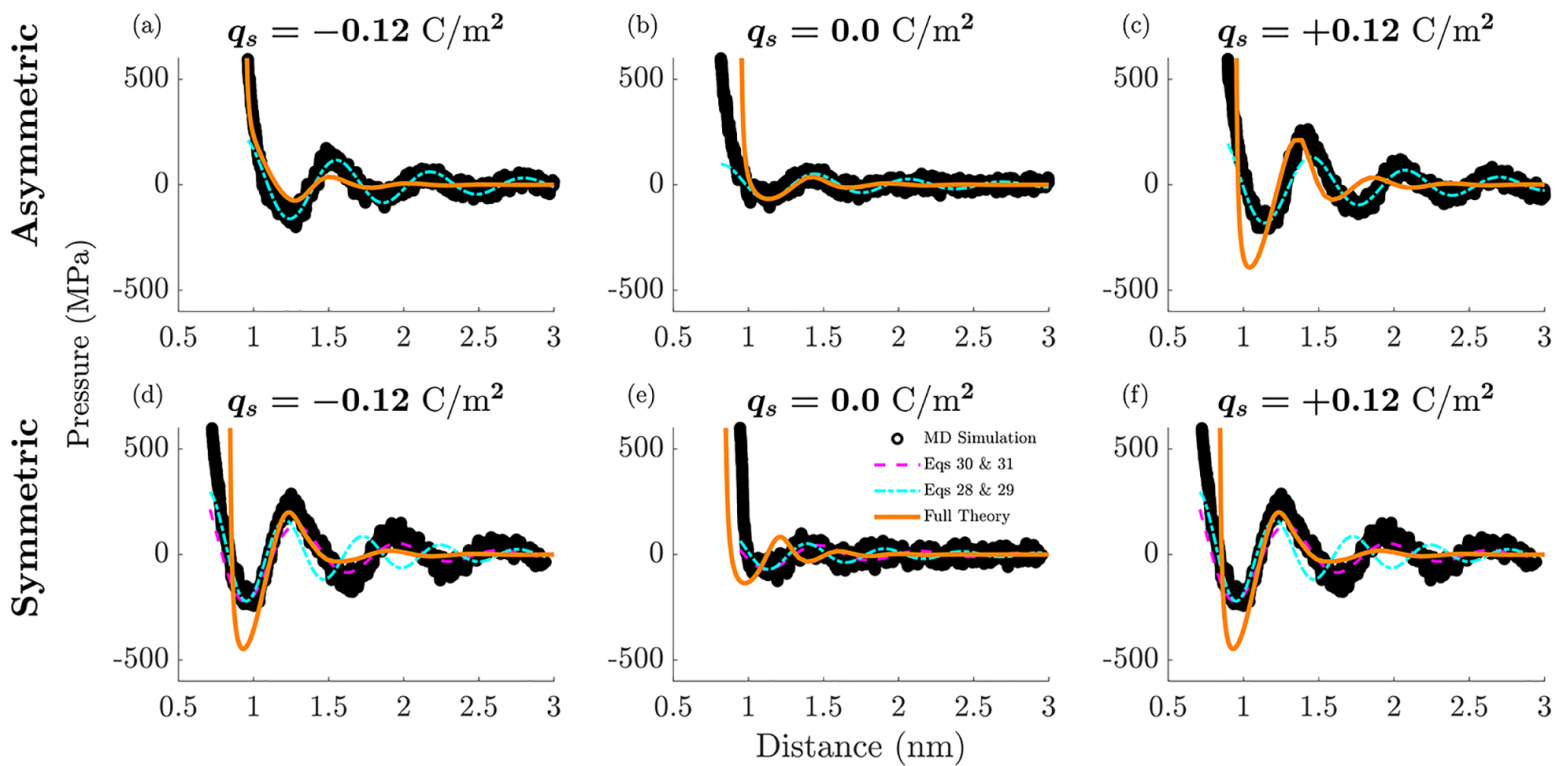
Disjoining pressure profiles. (a–c) Asymmetric system and (d–f) symmetric system for (a,d) negative, (b,e) uncharged, and (c,f) positive surfaces. The black markers (○) are the MD simulation data points. The solid orange lines are the full, nonlinear integro-differential theory. The other dashed and dash-dot lines are applications of the approximation in eq 14, where the parameters *P*_0_ and *z*_0_ are fit only to the first minimum. Here, the cyan dash-dot lines correspond to the analytical expressions for κ in eqs 28 and 29, while the magenta dashed lines plotted in (d–f) correspond to the definitions in eqs 30 and 31. These equations are derived in the framework of the linear response analysis.

Finally, the role of ionic asymmetry on structural forces is demonstrated in Figure 4. Pressure profiles are plotted as a function of the surface separation distance for the asymmetric (panels a–c) and symmetric (panels d–f) systems, where they are confined between negatively (panels a and d), uncharged (panels b and e), and positively charged surfaces (panels c and f). Additional results with varying surface charge magnitudes are presented in the SI. Comparing the theory to the simulations, overall, there is an agreement between the MD simulation pressure profiles (black markers ○ in Figure 4) and the full integro-differential results from the theory (solid orange lines in the top and bottom rows, the asymmetric and symmetric systems, respectively). This agreement is most pronounced at the smallest separation distances, where the pressure magnitudes are the most significant. The main discrepancy, as similarly noted already for the ionic density profiles, is that the oscillations decay more quickly for the theory than those that are observed in the simulations.

While the pressure profiles in Figure 4 appear to be similar for both systems, there are still significant differences with respect to surface charge magnitude and sign. Referring specifically to the simulated pressure profiles (black markers ○ in Figure 4), as expected, the amplitude of the pressure oscillations increases as the surface charge magnitude increases for both the asymmetric and the symmetric systems. However, for the asymmetric system, the pressure oscillation amplitudes are larger for the positive surface charge polarity, due to the larger anions accumulating between the positively charged surfaces. Meanwhile, as expected, the pressure profiles for the symmetric system do not depend on the sign of the surface charge density. In the asymmetric system, the period of oscillation is not affected by the electrode polarity or magnitude. This is because at small potential drops across the double layer, overscreening is always present, and the overscreening period and decay are determined by the diameter of the larger ion. For the symmetric system, the period of the pressure oscillations increases slightly at *q*_s_ = ±0.12 C/m^2^, yet it still remains on the scale of an individual ionic diameter.

The theory in general also captures these features, particularly the asymmetric pressure response of the asymmetric IL with changing surface polarity, as well as the oscillation period, being on the order of the largest ionic diameter (orange lines in Figure 4a–c).

#### 3.2.2 Linear Response Analysis

To gain further insight into the differences between the pressure profiles of the symmetric and asymmetric systems, we seek an approximate theoretical description in which the pressure profiles are represented by an exponentially decaying oscillating form,
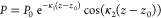
14where κ_1_ encodes the decay length of oscillations while κ_2_ encodes the period of oscillations. Approximating the theory at a linear response, the values for κ_1_ and κ_2_ are determined from the decay modes in the charge density, which is proportional to *c*_+_ – *c*_–_, and the total number density *c*_+_ + *c*_–_. While the equations presented thus far are generally nonlinear integro-differential equations, in the limit of small, slowly varying perturbations in linear response to the charge of the surfaces, we can derive analytical formulas to approximate the oscillatory decay of the charge and number density as a function of the IL properties. Here, we present the detailed derivation and analysis of the approximations involved in the linear response theory.

*Limit of small perturbations*: Equation 8 for small perturbations in the linear response can be approximated as
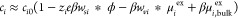
15The excess chemical potential can be linearized to give:
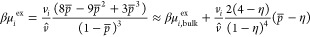
16where we have linearized the weighted filling fraction *p̅*, with reference value given by the bulk filling fraction, η. Therefore, if we assume linear perturbations of the bulk state, where *δf* = *f* – *f*_*b*_, we have the following coupled equations for the linearized Poisson equation and the ionic concentrations:
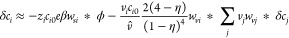
17
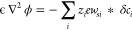
18

In the proceeding equations, we will analyze the decaying modes for these differential equations for the cases of (i) the symmetric IL system and (ii) the asymmetric IL system. We briefly comment on (iii) systems with only a small degree of size asymmetry. In each of these analyses, we will use the following differential approximation for the convolution integrals, derived by truncating the Fourier transform of the convolution operations for small perturbations,^22^ giving
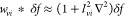
19
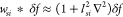
20where  and  are determined by the ionic size:
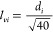
21
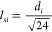
22where the numerical values of  and  are given directly from the mathematical form of the weighting functions.

*The symmetric case*: For the symmetric case, *w*_*si*_ = *w*_*s*_, *w*_*vi*_ = *w*_*v*_, and *v*_*i*_ = *v̂*. The linear response for the symmetric system was reported previously,^22^ but we again go through the process in order to draw contrast with the asymmetric case. For a 1:1 IL, we get the following:
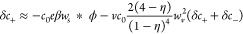
23
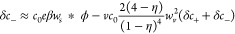
24
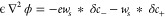
25If we sum the first two expressions and multiply by *v*, we get:
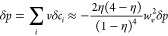
26where again, *p* = *v*∑_*i*_*c*_*i*_ is the local filling fraction. Next, if we subtract the first two expressions and substitute into the third equation, we get
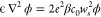
27

Here, we see that the equations for the potential and filling fraction at linear response are decoupled for the symmetric system. For the case where all functions become a function of *z* only, we can trial the solution *δf* = *A* exp(−*κz*) to find the values for the decaying modes, κ. For the equation governing the filling fraction, *δp* = *A*_*m*_ exp(−κ_*m*_*z*), (directly proportional to the total number density) the decay has both a real and imaginary part, where
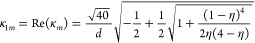
28
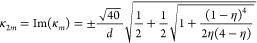
29The decay in number density is always oscillatory for nonzero filling fraction, since as η → 0, κ_1*m*_ ≈ κ_2*m*_ ≈ *d*^−1^(η/50)^−1/4^. On the other hand, as η → 1, the number density profile becomes purely oscillatory (without decay) with a period of oscillation close to the ion diameter.

Next, for the equation governing the electrostatic potential in the limit of high ionic concentration, with trial solution *δϕ* = *A*_*c*_ exp(−κ_*c*_*z*), the decaying mode has different real and imaginary components:
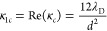
30
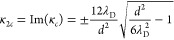
31where λ_D_ is the Debye length.

Even in linear response, we see that the two decaying modes will compete with one another in determining the overall disjoining pressure for the symmetric system. Both have similar nanometric decay ranges in the concentrated limit of ILs. At high charge density, the mode governing the decay of charge will dominate, κ_*c*_. At low charge density, the decay of charge is unimportant in the double layer structure, so the decay in the number density (and packing fraction), κ_*m*_ will dominate.

*The asymmetric case*: For the asymmetric system, it is more difficult to make analytical progress in solving for the decaying modes. First and foremost, the decay in number density (packing fraction) is coupled explicitly to the decay in charge density (potential). This fact arises because of the differences in the excess chemical potential between the different ions, which do not allow for the neat cancellations encountered with the symmetric system.

Further, even in solving the general problem for the decaying modes for each ionic species and the potential, analytical progress toward simple formulas is burdensome. For that reason, we will perform analysis for the limit of perfect asymmetry *d*_–_ ≫ (*d*_+_ ≈ 0) and high packing fraction. Then the governing linear response equations can be reframed as
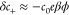
32
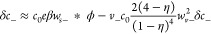
33
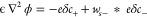
34By substitution for ϕ and *δc*_+_ into the equation for the potential, we get a single characteristic equation for the anion concentration decay:
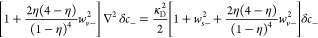
35Now, we can simplify this linearized expression by taking limits of κ_D_, in relation to the characteristic length scale of the gradients in concentration, which is the ionic diameter *d*_–_ at high concentration.

As a quick side note, the limit of *d*_–_ →0 where *w*_*s*–_ ≈ 1 gives the Debye–Huckel linearized form:
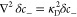
36so dilute electrolytes still have the Debye length (as it should be) as characteristic decay length, as long as the filling fraction η in the electrolyte is near zero.

If κ_D_*d*_–_ ≪ 1 and η ≠ 0, then we get the following leading sixth order differential equation for *δc*_–_:
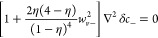
37valid near the interface. The more relevant limit to concentrated ILs is when κ_D_*d*_−_ → ∞, which at the leading order of eq 35 gives
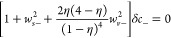
38For the purpose of simplicity, we can assume roughly that *w*_*s*–_ ≈ *w*_*v*–_. Next, at the filling fraction in the given parameter space, it is safely assumed that the term involving η dominates the differential equation at a large filling fraction, such that the form of the equation can be approximated as
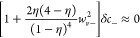
39In the limit of large filling fraction and perfect asymmetry, the longest decaying mode governing the decay of the ionic concentration can be approximated by κ_*m*_, again with its real and imaginary components:
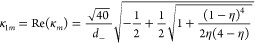
40
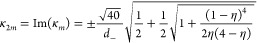
41Here, the asymmetric system has one main decaying mode, owing to the coupling of oscillations in charge and number density. As η → 1, the anion concentration profile becomes purely oscillatory with period roughly equal to the anion diameter, corresponding to a crystal of densely packed anions.

*Small degrees of asymmetry*: One important question is whether there is a smooth transition between the symmetric behavior and the asymmetric behavior depending on the extent of asymmetry. The degree of coupling between the charge density and total number density can be determined via the linear response equations. As a starting point, we consider the sum of the linear equations for the cation and anion, *δc*_+_ + *δc*_–_:
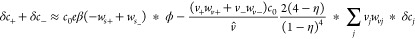
42The coupling of the charge density decay with the number density decay is controlled by the term containing ϕ. From this term, we observe that the system is decoupled when *w*_*s*+_ ≈ *w*_*s*–_, or in terms of the approximate differential operators, 1 + *d*_+_^2^/24∇^2^ ≈ 1+*d*_–_^2^/24∇^2^. From these relationships, we find that decoupling occurs when *d*_+_ ≈ *d*_–_, for symmetric ion sizes. Approximately, the extent of asymmetry can be quantified using the difference in differential operators, *w*_*s*–_ – *w*_*s*+_, assuming gradients on the order of the smallest length scale in the system, the smallest ion size (*d*_+_ in this case). Therefore, the system will reproduce perfect asymmetric behavior when
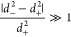
43and will reproduce perfect symmetric behavior when:
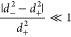
44Therefore, according to the linear response to small perturbations in the theory, systems with only slight asymmetry will behave similar to the symmetric system if they satisfy eq 44.

*Summary of linear response results*: In the linear response regime, we find that the charge density oscillations in the symmetric system are independent of the number density oscillations, as they are decoupled from each other. However, in the asymmetric system, in the linear response, consistent with our explanation for the results of the simulations and the full integro-differential theory, we find that the period (as well as the decay length) of oscillations in both charge and number density is determined by the diameter of the larger ion, and so the two are coupled to each other.

For the asymmetric system, in the limit of large filling fraction and perfect asymmetry, the longest decay mode in charge and number density can be approximately described by the definitions of κ_1*m*_ and κ_2*m*_ as derived above in eqs 40 and 41. Therefore, for the asymmetric system, this mode dominates the value of the disjoining pressure at all surface charges. The decay of oscillations in this high packing fraction limit is independent of the background permittivity and ionic charge. This is because in the regime of high packing, the structure and correlations in the system are determined majorly by the steric dense packing constraints, valid if κ_1*m*_ ≪ κ_D_.

For the symmetric system, as mentioned above, the ordering in charge and number density are decoupled. For this case, the number density decays with modes described by κ_1*m*_ and κ_2*m*_ in eqs 28 and 29, similarly to eqs 40 and 41, since these formulas are generally applicable for the decay in number density in a dense fluid at a hard wall. In contrast, the charge density decays as given by the definitions for κ_1*c*_ and κ_2*c*_ in the highly concentrated limit by eqs 30 and 31. For the symmetric system at low surface charge, the oscillations in charge are less pronounced than the oscillation in the number density, so the decay in the number density (and filling fraction), κ_*m*_, will dominate in determining the pressure profile. Alternatively, at high surface charge, the mode governing the decay of charge, κ_*c*_, will dominate. One could expect similar trends for ions that are only approximately symmetric in size, with only a small degree of asymmetry. Therefore, as *v*_–_ → *v*_+_, there is a transition between the perfectly asymmetric and the perfectly symmetric behaviors, as analyzed in the linear response behavior above.

We present our findings for both the symmetric and asymmetric systems in Figure 4, where the approximations corresponding to eq 14 with κ_*m*_ as given in eqs 28 and 29, and κ_*c*_ as given in eqs 30 and 31 are plotted, manually fitting the point of the first minimum of the simulation data with *P*_0_ and *z*_0_, but keeping all other analytical formulas above. The decay decrement, κ_*m*_, governed by the filling fraction is plotted with the dashed-dot cyan lines, while the decay decrement, κ_*c*_, governed by the decay in charge is plotted with the dashed magenta lines.

For the asymmetric system (panels a–c), as mentioned above, κ_*m*_ describes the decay at all surface charges. Therefore, in Figure 4, one can observe that the dashed-dot cyan lines fitted to the first minimum compare almost perfectly at all surface charges to the pressure oscillations found in the simulations.

For the symmetric system, however, the decaying mode given by κ_*m*_, plotted with the dashed-dot cyan lines, only dominates at zero surface charge (panel e). In contrast, since the decay in pressure oscillations at high electrode charges such as *q*_*s*_ = ± 0.12 C/m^2^ is dominated by the decrement, κ_*c*_, the plotted dashed magenta lines in Figure 4, panels d and f, match better with the pressure oscillations found in the simulations at these high surface charges (one can see how the dashed-dot cyan line for κ_*m*_ is in offset and does not describe well the simulated pressure oscillations).

Seeing as how the approximate equations of the linear response theory describe quite accurately the decay of the simulated pressure, we can now compare and validate quantitatively the periodicities of the pressure oscillations displayed in Figure 4. In the simulations, the period of the first oscillation (distance from first to second minimum) in the asymmetric system for the surface charges of *q*_*s*_ = −0.12, −0.06, 0, +0.06, and +0.12 C/m^2^ are 0.59, 0.62, 0.57, 0.60, and 0.61 nm, respectively. This compares well to the result from the linearized formula for κ_2*m*_ in eq 29, which predicts a period of 0.62 nm for the asymmetric system. This value is essentially the effective largest ionic diameter *d*_*i*_ ≈ 0.9σ_*i*_, where σ_–_ = 0.7 nm (our criterion that takes into account the overlap of ions in the simulations), showing numerically that the periodicity of oscillations in asymmetric systems at all surface charges is determined by the diameter of the larger ion. For the symmetric system, the simulated periods of the first oscillation for *q*_*s*_ = 0, ±0.06, and ±0.12 C/m^2^ are 0.53, 0.58, and 0.66 nm, respectively. The periodicity values at low surface charges are given by κ_2*m*_ in eq 29, which predicts a period of 0.52 nm (again, *d*_*i*_ ≈ 0.9σ_*i*_, where σ_±_ = 0.58 nm). At high surface charge densities, the periodicity values are given by κ_2*c*_ in eq 31, which predicts a period of 0.68 nm, numerically showing the increase in the oscillation period with surface charge magnitude in this case.

We note that while in previous experimental measurements of surface forces in IL, the oscillation period has been described in terms of the ion pair diameter,^8,9,47,48^ the oscillation period in the simulations and the theory presented here, at the high concentration limit, more closely matches the diameter of the largest ion, in all cases, multiplied by the scalar prefactor that is close to 1.

## 4 Conclusions

All in all, the change in the oscillatory decay as a function of surface charge underlies a major difference between charge screening in concentrated asymmetric systems compared to concentrated symmetric ones. In asymmetric systems, the decay modes in charge and number density are coupled to each other, and therefore give the same decay mode. For the symmetric system, the two are decoupled. This essentially leads to differences in the microscopic ionic concentration profiles in nanoconfinement as a function of electrode charge magnitude and polarity, and ultimately to an observable difference in the disjoining pressure profile. Our unraveled findings presented here provide some generalizable insights into the detailed behaviors of such concentrated ionic systems. These insights, therefore, may be taken into consideration in terms of the analytical expressions that are derived in our work, and can help interpret results of future experimental data of structural forces in ionic liquids, as well as simulations.

To summarize, the main novel scientific contribution of our work is our proposed continuum theory which describes well the density, charge distributions, and structural forces of ILs in nanoscale confinements and the effect of surface polarization on these quantities. Through application of this theory, we can relate the oscillation periodicity and decay of the molecular structuring and charge density oscillations in nanoconfinement to the physical properties of the IL, including the bulk ionic density, the ionic sizes, the temperature, and the background permittivity. While the MD simulations and theory profiles do not match perfectly, both approaches predict layered structures that lead to structural forces at small separation distances. Both simulations and theory also recover the main features of screening in asymmetric ILs, which are not present in symmetric ILs. Those include the variation of force amplitudes depending on the surface charge polarity, the “preferential adsorption” of smaller ions at zero electrode charge, and the coupling of charge and number density oscillations in systems at such high ionic concentration. Therefore, on the basis of our findings, we conclude that the ionic size asymmetry is an important ingredient in describing ILs at the nanoscale.

## Supporting Information Available

Additional simulation details and results with varying theory parameters (PDF)

## Glossary of Symbols

σ_*i*_: LJ Parameter for the *i*,*i* interaction
*F*_*L*_: normal force
*L*: plate separation distance
*c*_0_: bulk ionic concentration
ϵ: background permittivity
ϵ_0_: permittivity of free space
*T*: absolute temperature
*q*_*s*_: surface charge density
Helmholtz free energy
ideal contribution to free energy
excess contribution to free energy
electrostatic contribution to free energy
*k*_*B*_: Boltzmann constant
*c*_*i*_: concentration of species *i*
*p̅*: weighted volumetric filling fraction
*v̂*: weighted volume
*v*_*i*_: ionic volume of species *i*
*R*_*i*_: effective hard-sphere ionic radius of species *i*
*w*_*v*,*i*_: volumetric weighting function
η: bulk filling fraction
*c*_*i*,0_: bulk ionic concentration of species *i*
ϕ: electrostatic potential
ρ̅_*e*_: homogenized charge density
*z*_*i*_: valency of ion *i*
*e*: elementary charge
*w*_*s*,*i*_: surface weighting function
β: inverse thermal energy
μ_*i*_^ex^: excess chemical potential
μ_*i,b*_^ex^: bulk excess chemical potential
*R*_*s*_: surface atom radius
*d*_*i*_: effective hard-sphere ionic diameter of species *i*
*U*_LJ_: LJ potential
*P*: pressure
*A*: area
Ω: grand potential
*P*_∞_: reference pressure as *L* →∞
*Q*_cu_: cumulative charge function
*P*_0_: pressure at first minimum
*z*_0_: separation distance at first minimum
κ_1_: inverse decay length of pressure
κ_2_: oscillation frequency of pressure
*δf*: linear perturbation function
*f*: local value of function
*f*_*b*_: bulk reference value of function
length scale for differential approximation of *w*_*v*,*i*_
length scale for differential approximation of *w*_*s*,*i*_
κ_1*m*_: inverse decay length of total number density
κ_2*m*_: oscillation frequency of total number density
κ_1*c*_: inverse decay length of charge density (symmetric system)
κ_2*c*_: oscillation frequency of charge density (symmetric system)
λ_D_: Debye length
κ_D_: inverse Debye length
