# Supplementary material for: Structural Forces in Ionic Liquids: The Role of Ionic Size Asymmetry
Source: J Phys Chem B. 2022 Feb 8;126(6):1242–53. doi: 10.1021/acs.jpcb.1c09441 (PMC9007453; doi:10.1021/acs.jpcb.1c09441)
Supplement: Supplementary file 1 — jp1c09441_si_001.pdf [file jp1c09441_si_001.pdf]

Supporting Information for Structural Forces in Ionic Liquids: The Role of Ionic Size Asymmetry

J. Pedro de Souza,[†,⊥] Karina Pivnic,[‡,⊥] Martin Z.Bazant,[†,¶] Michael Urbakh,[‡] and Alexei A. Kornyshev[*,§,‖]

†*Department of Chemical Engineering, Massachusetts Institute of Technology, Cambridge, MA, USA*

‡*School of Chemistry, The Sackler Center for Computational Molecular and Materials Science, Tel Aviv University, Tel Aviv 6997801, Israel*

¶*Department of Mathematics, Massachusetts Institute of Technology, Cambridge, MA, USA*

§*Department of Chemistry, Molecular Sciences Research Hub, Imperial College London, W12 0BZ 2AZ London, United Kingdom*

‖*Thomas Young Centre for Theory and Simulation of Materials, Imperial College London, South Kensington Campus, London SW7 2AZ, United Kingdom*

⊥*These authors contributed equally to this work.*

E-mail: a.kornyshev@imperial.ac.uk

1 Additional simulation details

We perform Grand Canonical Molecular Dynamics (GCMD) simulations[1–3] to study the role of the size asymmetry of ionic liquids (ILs) on their screening behavior and the resulting structural forces. To model the ILs, we use a minimal model of the ions for which they are represented as a 1:1 mixture of oppositely and singly charged Lennard-Jones (LJ) spheres,[4,5] as shown in Figure 1 of the main text. Such simulations of generic features of ILs provided

a clear insight into the effects of overscreening and crowding at surfaces and in nanogaps,[4–7] and we adopt a similar approach here. The ions in the simulation box interact through short-range LJ, and coulombic potentials, where the size asymmetry of the ions is controlled by adjusting their diameters through the LJ parameter σ_{ij} for species i interacting with species j, or σ_i when $i = j$. The ion sizes that we consider here for the asymmetric system are $\sigma_- = 0.7$ nm and $\sigma_+ = 0.35$ nm, and the ion size for the symmetric system is $\sigma_- = \sigma_+ = 0.58$ nm, such that the filling fraction is approximately equal in both systems, making $\sum_i \sigma_i^3 c_0$ unchanged. The simulation box, filled with either the asymmetric or the symmetric ions, contains a mixture consisting of about 5,478 cations and 5,478 anions (depending on surface charge the number of cations and anions in the simulations box was slightly changed to preserve the overall electroneutrality), which leads to a concentration of both cations and anions of $c_0 = 4.586$ M.

Two parallel solid plates in the x–y plane mimicking mica surfaces are immersed in the bulk of IL (see Figure 1 of the main text). In constructing the surfaces, each plate includes 2,838 LJ spheres, with the FCC (111) plane in contact with the confined liquid, and a lattice parameter of 0.36 nm. This structure resembles the effective closed packed structure of the hexagonal patterns of SiO_4 units in a cleaved plane of mica,[8] as it was detected previously by AFM experiments.[9] The effective LJ diameter of the spheres in the plates is set to 0.3218 nm.[5] The LJ potential interaction strength of the spheres in each plate is set to 500 kJ/mol. This interaction strength ensures that a significant deformation of the plates is prevented.[5] The LJ spheres located in different plates interact with each other through electrostatic interactions only, unless the separation between them is shorter than the LJ cutoff, taken here as 1.8 nm.

In the simulations, we assign partial charges to the surface atoms of the plates that are in contact with the confined liquid. The plates thus serve as electrodes and provide the electrostatic potential in the simulation box. The surface charge density of each plate, q_s, is varied between -0.12 and +0.12 C/m^2. In experiments, when dealing with conducting

surfaces, one controls the potential to which the charge distribution on the surfaces is adjusted. However, to avoid excessive computational burden, our simulations are performed using the fixed charge method, with fixed partial charges assigned to the surface atoms in the plates, hence allowing a control of the surface charge density. For each surface charge density, q_s, one can calculate the surface potential with respect to the bulk of IL, as well as the potential distribution inside the nanogap. The electrostatic potentials, which emerge in our simulations, lie below the range of one Volt, in accordance with the typical range of q_s estimated in experiments.[6,10]

Besides the Coulombic interactions, the spheres in the solid plates interact with the ions through short range LJ interactions, and so do the ions with one another. The ion-ion interaction is set to $\epsilon_{ii} = 0.25$ kJ/mol, the ion-plate interaction to $\epsilon_{ip} = 1.5$ kJ/mol, and the sphere sizes of both the plates and the ions are set by the mixing rule $\sigma_{ij} = (\sigma_i + \sigma_j)/2$.

Structural forces are simulated by slowly approaching the plates towards one another in the z direction using a very low constant and finite velocity of 0.1 m/s, such that it is close to the quasi-static limit. In this way, when the plates approach one another, liquid molecules are squeezed out of the confining region between the surfaces, and the resulting average forces on the plates are monitored as a function of the distance between them. By using this method, the whole range of inter-plate distances can be accessed, reaching both stable and unstable states, and the full pressure profiles are produced.[11,12] While in experiments, due to the force measuring technique, the structural forces are detected as discontinuous force-displacement curves which result from load induced-instabilities leading to the jumps of the pushed plates,[10,13] here we emulate the full-ranged structural forces in order to compare the disjoining pressure profiles to those calculated in the framework of the continuum theory.

The ionic density profiles between the surfaces are computed from an additional set of simulations, where we apply identical constant normal loads, to both plates, which match the forces found in the stable state regions of the disjoining pressure profiles. In those simulations, for each applied normal load, the liquid molecules are squeezed out of the confining region as

the plates approach one another until they reach their equilibrium positions. Upon reaching a fixed equilibrium separation distance between the plates, we then produce and plot the density profiles in the corresponding confined region.[11]

1.1 Simulation Methods

Our computations are performed using the molecular dynamics code, Gromacs v. 4.6.3.[14] In our simulations, we use the Grand Canonical Molecular Dynamics (GCMD) simulation method.[1–3] In this method, the confined region between plates is simulated explicitly by setting the plates in the bulk fluid. The confined region between the plates is then in contact with a reservoir fluid; hence, the chemical potential in that region is determined by the chemical potential of the reservoir by allowing the IL to enter or leave it as a response to changes in the normal pressure, temperature, or surface charge.[1] The electroneutrality of the simulated system is preserved for all values of surface charge densities.

All the simulations are performed at 600 K by rescaling the velocities[15] of the plates atoms. This serves to maintain the temperature of the IL ions, which are not coupled to an explicit thermostat. The charged LJ-spheres are simulated in such elevated temperature since at room temperature dense plasma of the spheres freezes out. The system is also coupled to a pressure barostat, set at 1 bar pressure, via the anisotropic Berendsen algorithm,[16] which is applied to the x and z directions. Periodic boundary conditions are imposed in all directions. The long-range electrostatic interactions are computed using the 3D–Particle Mesh Ewald summation method using "tin-foil" boundary conditions. All Coulomb interactions in the system are screened by an effective dielectric constant of $\epsilon = 2\epsilon_0$, where ϵ_0 is the dielectric constant of free space, which accounts for electronic polarizability of the liquid.

To produce the disjoining pressures, the simulations are performed in two steps. In the first step, the system including the liquid confined between the fixed plates located at a distance of 6 nm from each other is equilibrated during 8 ns. In the second simulation step, which lasts for about 80 ns, the plates are slowly approached towards one another using a

very low constant and finite velocity of 0.1 m/s, such that it is close to the quasi-static limit. This step is essentially the production run, where the force is monitored as a function of the distance between the plates, to ultimately obtain the disjoining pressure profiles. To produce the ionic density profiles, an additional set of simulations are performed, in three steps, using a different approach. The first step in those simulations is essentially the same equilibration step run in the simulations for the disjoining pressures. In the second simulation step, lasting for about 20 ns, identical constant normal loads, equivalent to the forces found in the stable state regions of the disjoining pressure profiles, are applied to both plates, and the distances between the plates are recorded upon reaching equilibrium. Then, in a third simulation step, the plates which are located at a fixed separation distance are further equilibrated for an additional 10 ns, as a production run, to ultimately calculate and obtain the ionic density profiles confined between the corresponding fixed plate separation distance. The Leap-Frog algorithm is used in all the simulations to integrate the equations of motion, using a time step of 2 fs, and configurations are saved every 6 ps for the structural analyses.

2 Determining the Theory Parameters

Here, we include all the results, including all pressure plots and all ionic density profiles, depending on the relationship between the theoretical ionic diameter value, d_i, and the LJ simulation parameters, σ_i. Below, we present and contrast two choices for the relationship between d_i and σ_i: $d_i = 0.9\sigma_i$ and $d_i = 1.0\sigma_i$.

The figures are organized as follows:

- Figures S1-S5 show the ionic density for varying surface charge densities and separation distances for the asymmetric system with $d_i = 0.9\sigma_i$.
- Figures S6-S8 show the ionic density for varying surface charge densities and separation distances for the symmetric system with $d_i = 0.9\sigma_i$.
- Figures S9-S13 show the ionic density for varying surface charge densities and separation distances for the asymmetric system with $d_i = 1.0\sigma_i$.
- Figures S14-S16 show the ionic density for varying surface charge densities and separation distances for the asymmetric system with $d_i = 1.0\sigma_i$.
- Figures S17-S18 show the disjoing pressure profiles for the asymmetric and symmetric systems, respectively, for $d_i = 0.9\sigma_i$.
- Figures S19-S20 show the disjoing pressure profiles for the asymmetric and symmetric systems, respectively, for $d_i = 1.0\sigma_i$.

Further, the plots are organized into subsections with descriptive titles that distinguish these differences.

Overall, while the profiles are qualitatively similar for either choice of the relationship between d_i and σ_i, we can make some general statements comparing and contrasting them. First, the magnitude of the pressures for $d_i = 0.9\sigma_i$ is much closer to the simulation results than for $d_i = 1.0\sigma_i$. This trend might be expected, given that the softness of the LJ potential

corresponds to the occurrence of the 'overlap' of ions, which ultimately reduces the effective packing fraction and thus decreases the disjoining pressure magnitude. On the other hand, the ionic density and cumulative charge density plots are slightly improved for $d_i = 1.0\sigma_i$ compared to $d_i = 0.9\sigma_i$. This trend is more nuanced to rationalize, but mainly corresponds to increasing the theoretically-predicted overscreening magnitude with increasing d_i, bringing the theory predictions slightly closer to the simulations (for all systems tested, the theory predicts less overscreening than the simulations at large separation distances). For the purposes of this study, we chose $d_i = 0.9\sigma_i$ as the baseline choice presented in the main text, as it captured the magnitudes and trends in the charge density oscillations and disjoining pressure profiles.

2.1 Asymmetric Ionic Density Comparisons, $d_i = 0.9\sigma_i$

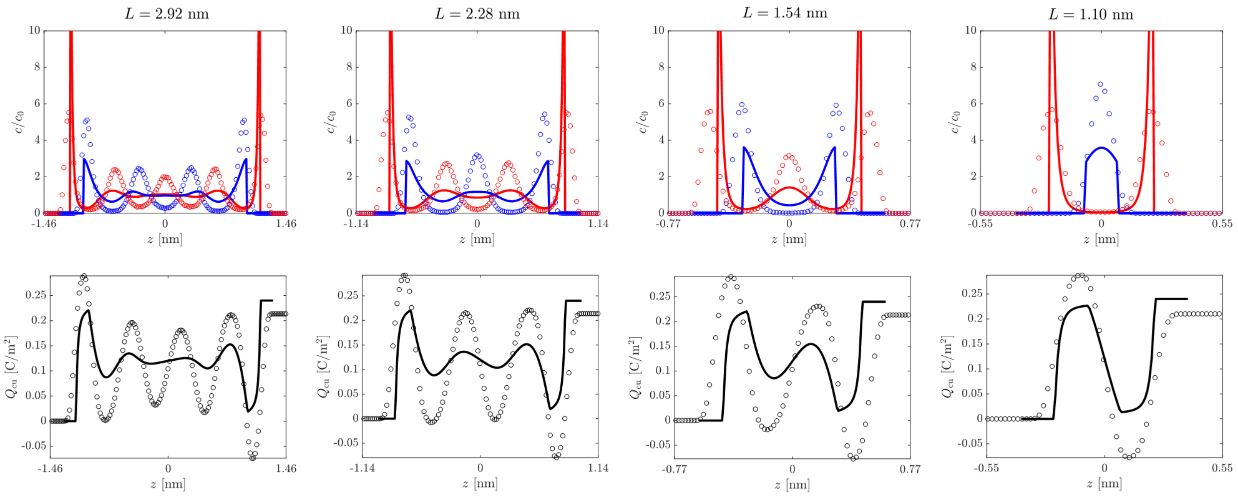

Figure S1: ($q_s = -0.12$ C/m^2, $d_i = 0.9\sigma_i$, Asymmetric) Charge and ion density profiles in asymmetric ILs between the charged plates. Markers, (○): simulations; Lines, (—): theory. Color coding: (—) - anions, (—) - cations.

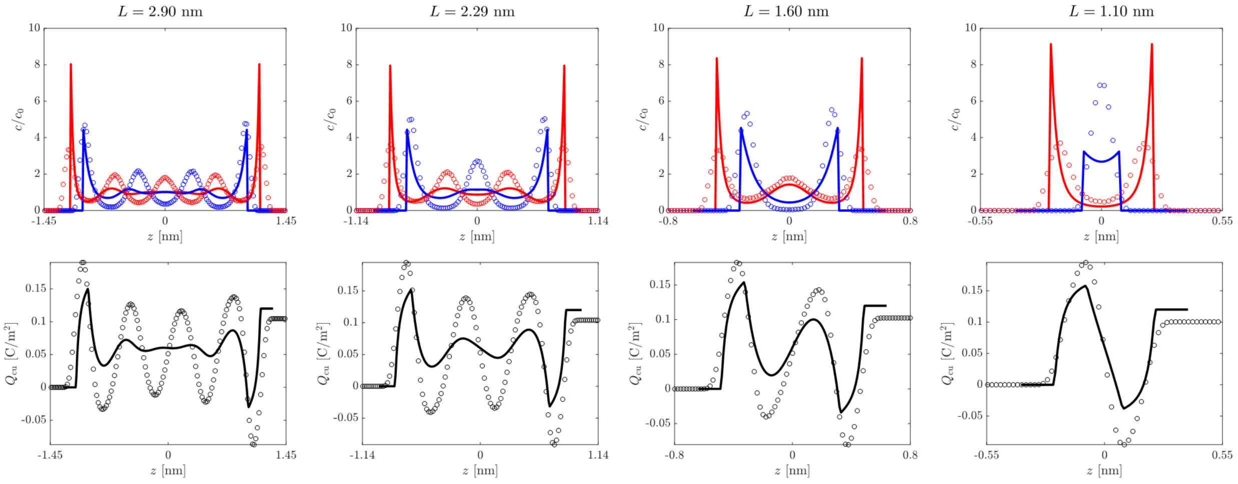

Figure S2: ($q_s = -0.06$ C/m^2, $d_i = 0.9\sigma_i$, Asymmetric) Charge and ion density profiles in asymmetric ILs between the charged plates. Markers, (∘): simulations; Lines, (—): theory. Color coding: (—) - anions, (—) - cations.

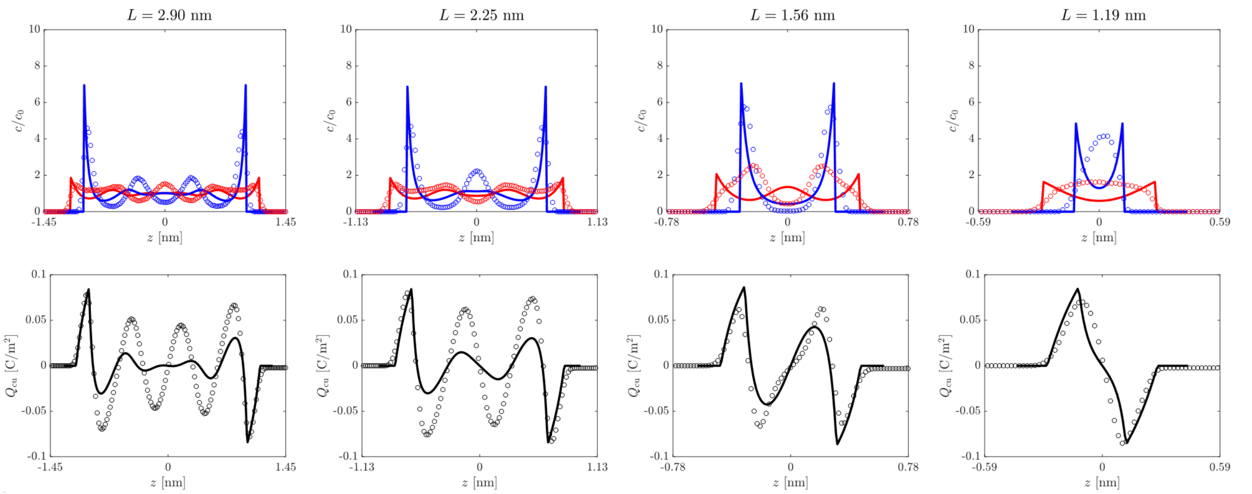

Figure S3: ($q_s = 0$ C/m^2, $d_i = 0.9\sigma_i$, Asymmetric) Charge and ion density profiles in asymmetric ILs between the charged plates. Markers, (∘): simulations; Lines, (—): theory. Color coding: (—) - anions, (—) - cations.

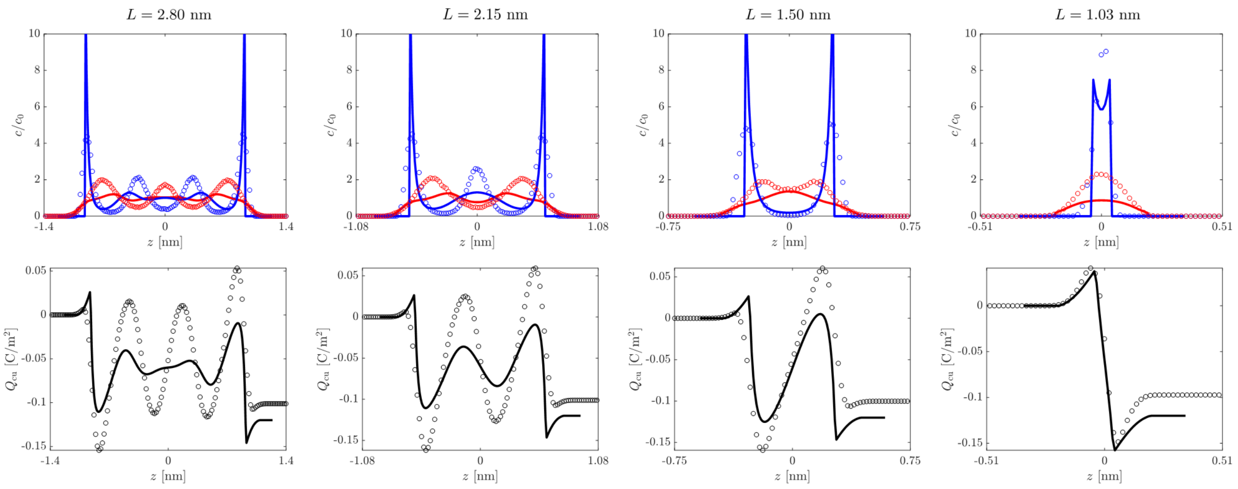

Figure S4: ($q_s = +0.06$ C/m^2, $d_i = 0.9\sigma_i$, Asymmetric) Charge and ion density profiles in asymmetric ILs between the charged plates. Markers, (○): simulations; Lines, (—): theory. Color coding: (—) - anions, (—) - cations.

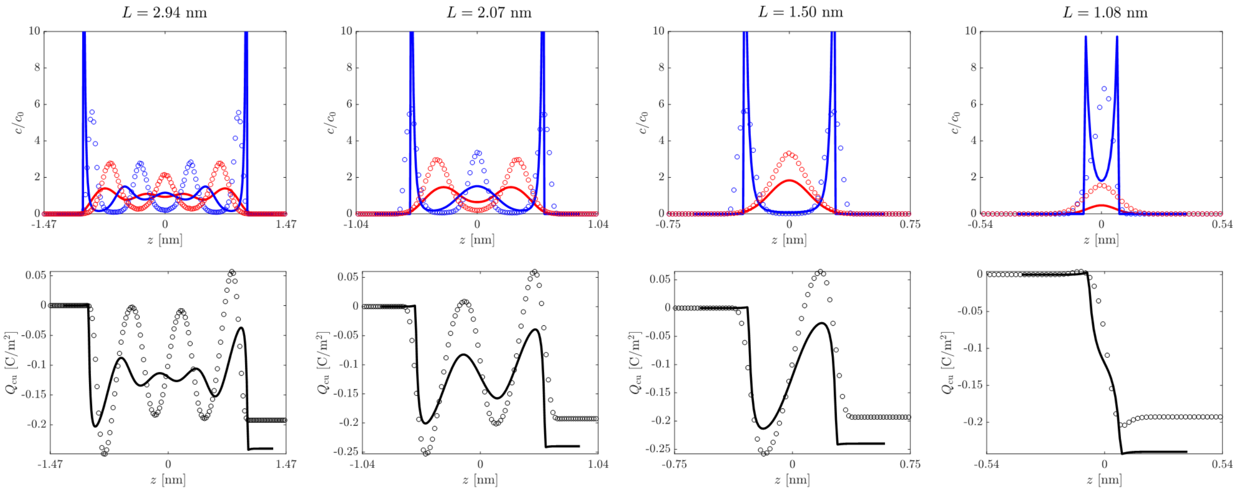

Figure S5: ($q_s = +0.12$ C/m^2, $d_i = 0.9\sigma_i$, Asymmetric) Charge and ion density profiles in asymmetric ILs between the charged plates. Markers, (∘): simulations; Lines, (—): theory. Color coding: (—) - anions, (—) - cations.

2.2 Symmetric Ionic Density Comparisons, $d_i = 0.9\sigma_i$

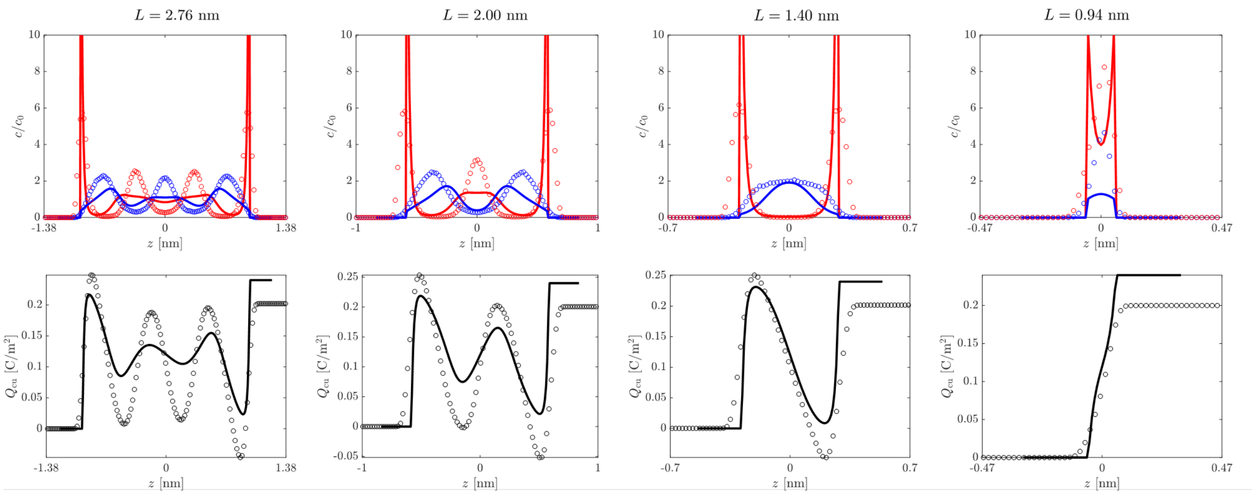

Figure S6: ($q_s = -0.12$ C/m^2, $d_i = 0.9\sigma_i$, Symmetric) Charge and ion density profiles in symmetric ILs between the charged plates. Identical profiles are found for $q_s = +0.12$ C/m^2, up to the identity of the symmetric ions. Markers, (○): simulations; Lines, (—): theory. Color coding: (—) - anions, (—) - cations.

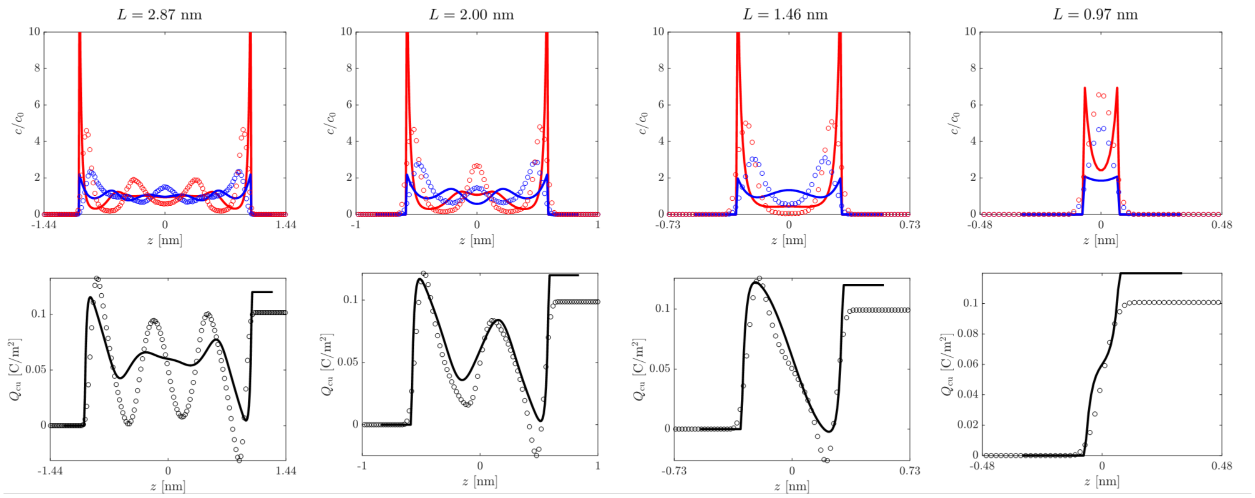

Figure S7: ($q_s = -0.06$ C/m^2, $d_i = 0.9\sigma_i$, Symmetric) Charge and ion density profiles in symmetric ILs between the charged plates. Identical profiles are found for $q_s = +0.06$ C/m^2, up to the identity of the symmetric ions. Markers, (○): simulations; Lines, (—): theory. Color coding: (—) - anions, (—) - cations.

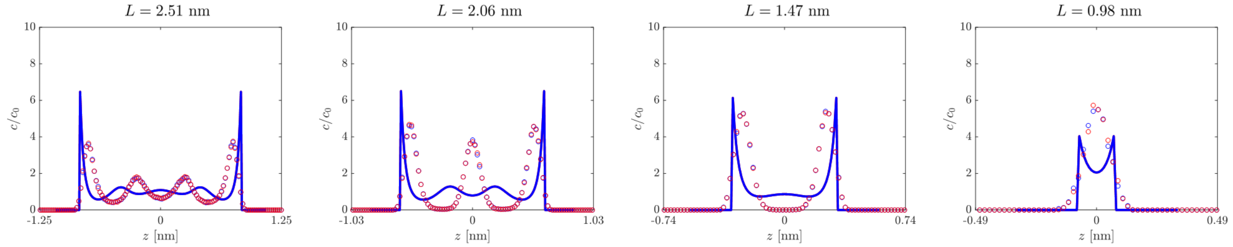

Figure S8: ($q_s = 0$ C/m^2, $d_i = 0.9\sigma_i$, Symmetric) Charge and ion density profiles in symmetric ILs between the charged plates. Markers, (○): simulations; Lines, (—): theory. Color coding: (—) - anions, (—) - cations.

2.3 Asymmetric Ionic Density Comparisons, $d_i = 1.0\sigma_i$

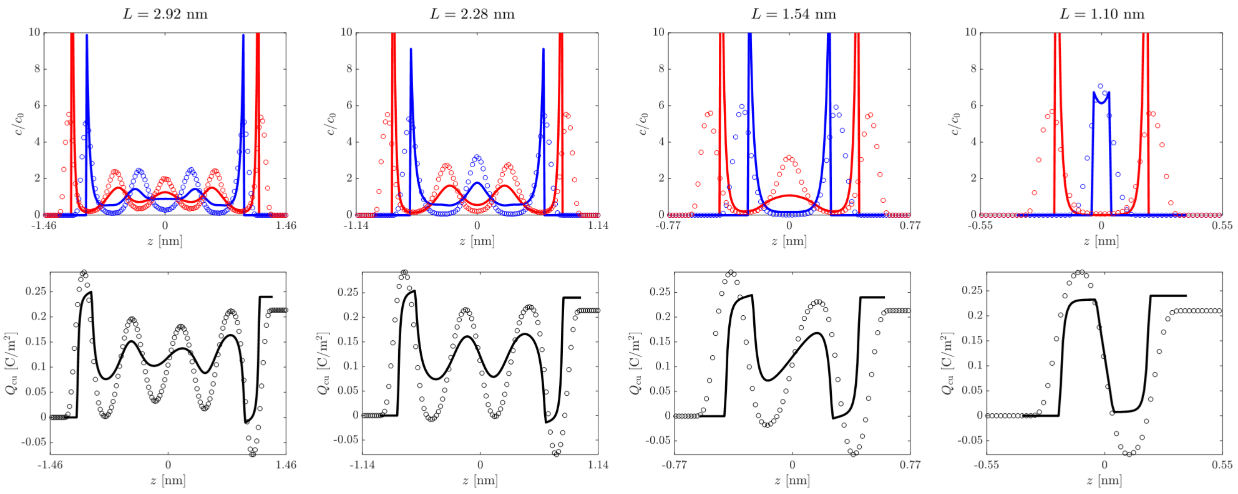

Figure S9: ($q_s = -0.12$ C/m^2, $d_i = 1.0\sigma_i$, Asymmetric) Charge and ion density profiles in asymmetric ILs between the charged plates. Markers, (○): simulations; Lines, (—): theory. Color coding: (—) - anions, (—) - cations.

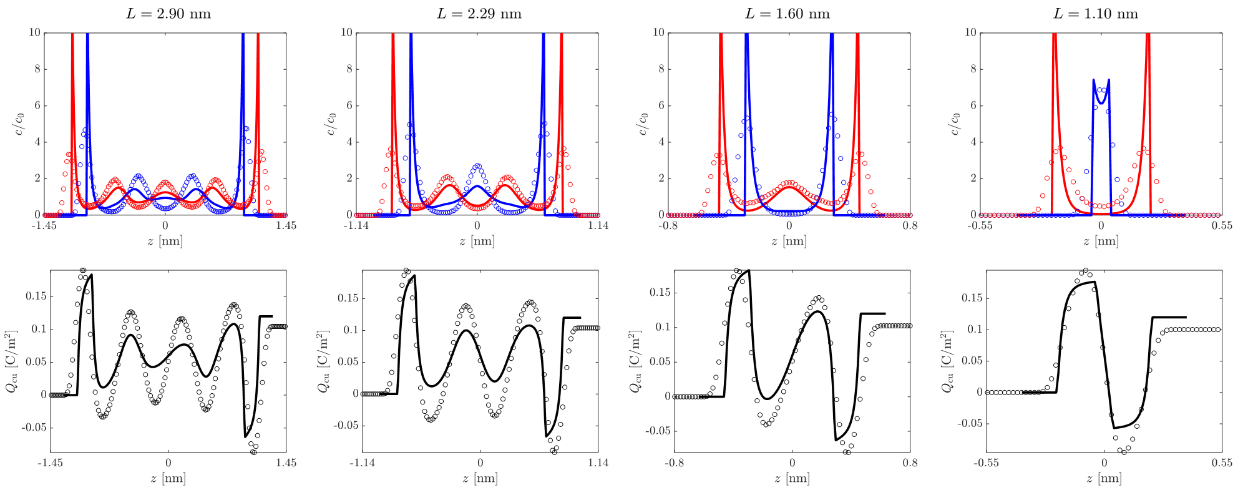

Figure S10: ($q_s = -0.06$ C/m^2, $d_i = 1.0\sigma_i$, Asymmetric) Charge and ion density profiles in asymmetric ILs between the charged plates. Markers, (○): simulations; Lines, (—): theory. Color coding: (—) - anions, (—) - cations.

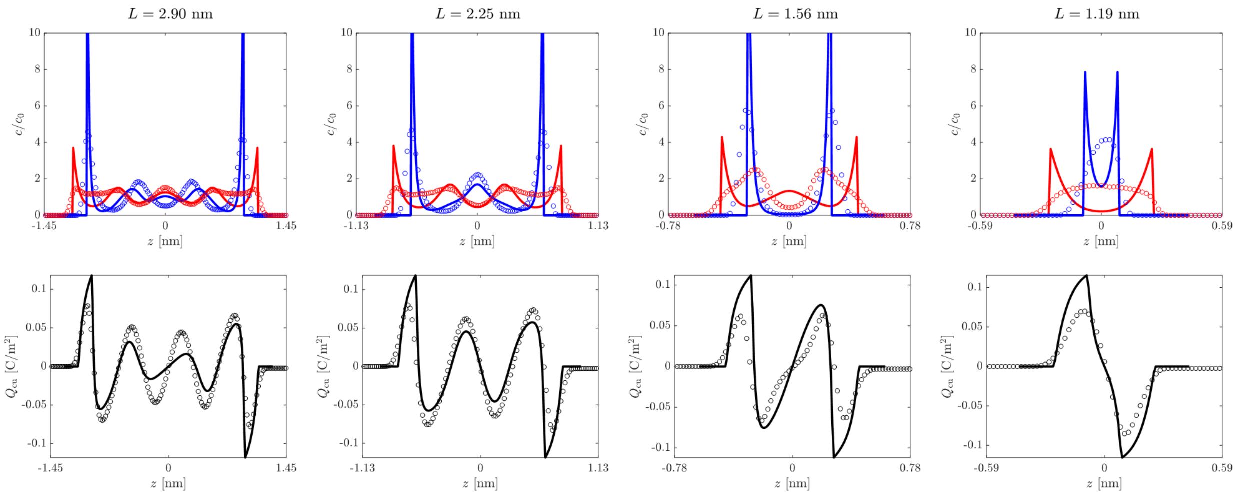

Figure S11: ($q_s = 0$ C/m^2, $d_i = 1.0\sigma_i$, Asymmetric) Charge and ion density profiles in asymmetric ILs between the charged plates. Markers, (∘): simulations; Lines, (—): theory. Color coding: (—) - anions, (—) - cations.

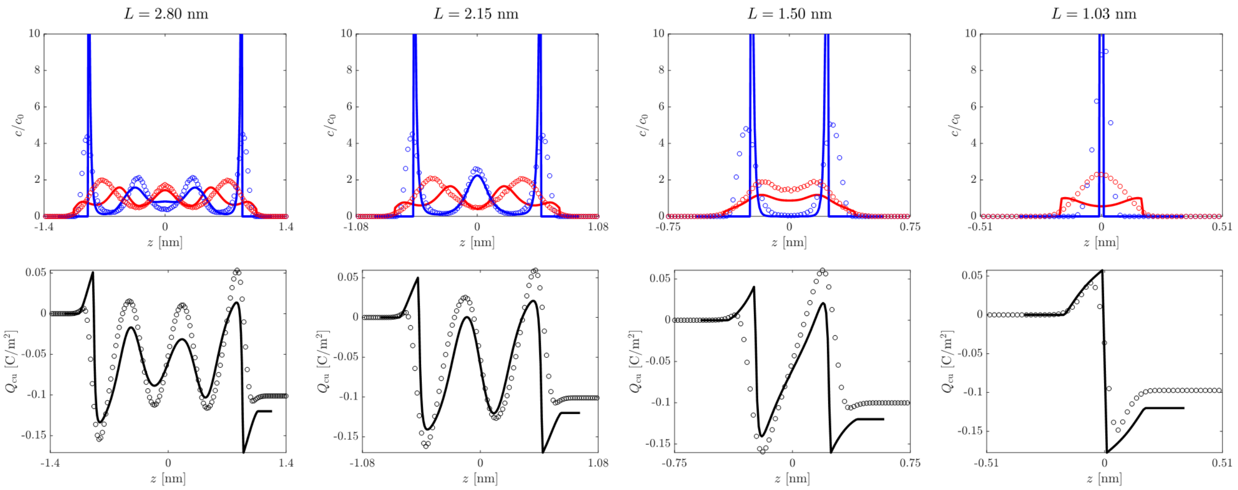

Figure S12: ($q_s = +0.06$ C/m^2, $d_i = 1.0\sigma_i$, Asymmetric) Charge and ion density profiles in asymmetric ILs between the charged plates. Markers, (∘): simulations; Lines, (—): theory. Color coding: (—) - anions, (—) - cations.

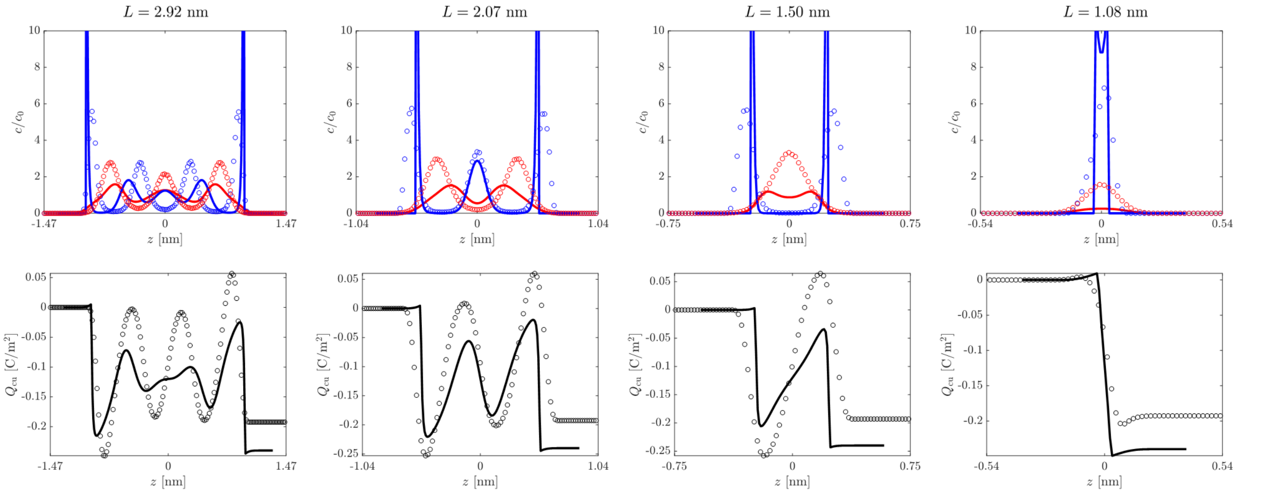

Figure S13: ($q_s = +0.12$ C/m^2, $d_i = 1.0\sigma_i$, Asymmetric) Charge and ion density profiles in asymmetric ILs between the charged plates. Markers, (∘): simulations; Lines, (—): theory. Color coding: (—) - anions, (—) - cations.

2.4 Symmetric Ionic Density Comparisons, $d_i = 1.0\sigma_i$

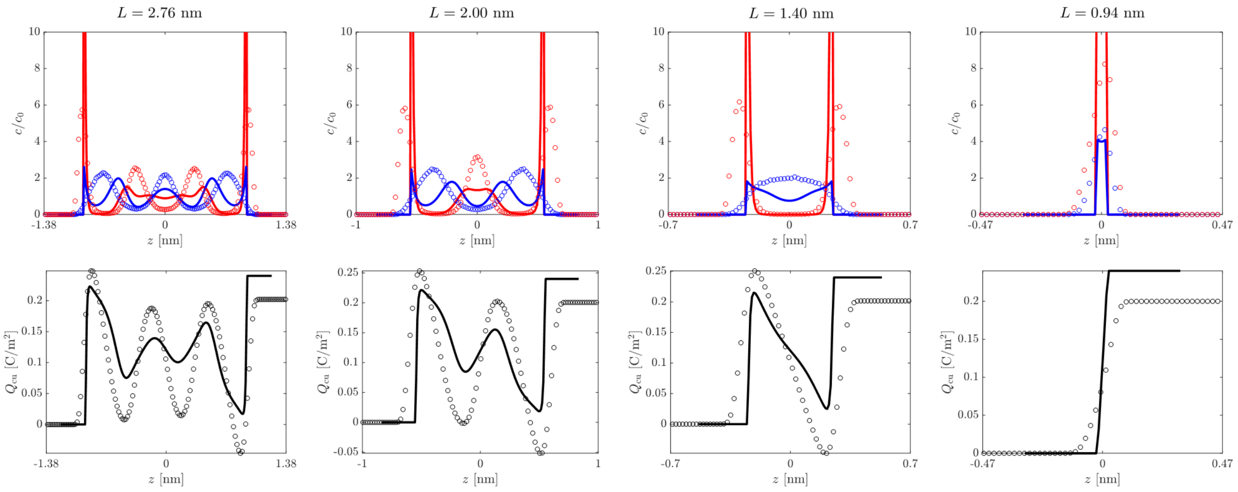

Figure S14: ($q_s = -0.12$ C/m^2, $d_i = 1.0\sigma_i$, Symmetric) Charge and ion density profiles in symmetric ILs between the charged plates. Identical profiles are found for $q_s = +0.12$ C/m^2, up to the identity of the symmetric ions. Markers, (∘): simulations; Lines, (—): theory. Color coding: (—) - anions, (—) - cations.

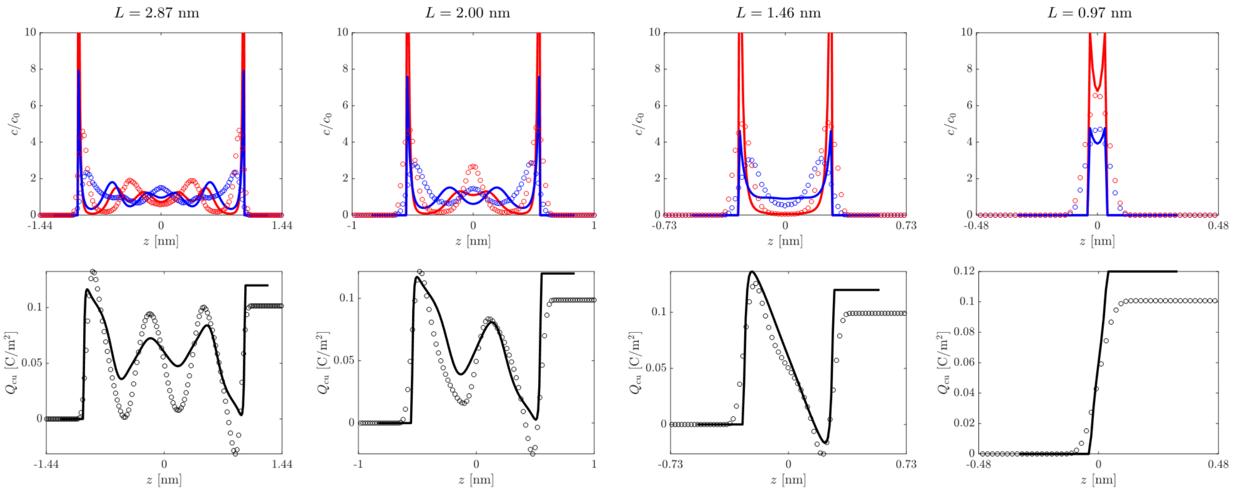

Figure S15: ($q_s = -0.06$ C/m^2, $d_i = 1.0\sigma_i$, Symmetric) Charge and ion density profiles in symmetric ILs between the charged plates. Identical profiles are found for $q_s = +0.06$ C/m^2, up to the identity of the symmetric ions. Markers, (○): simulations; Lines, (—): theory. Color coding: (—) - anions, (—) - cations.

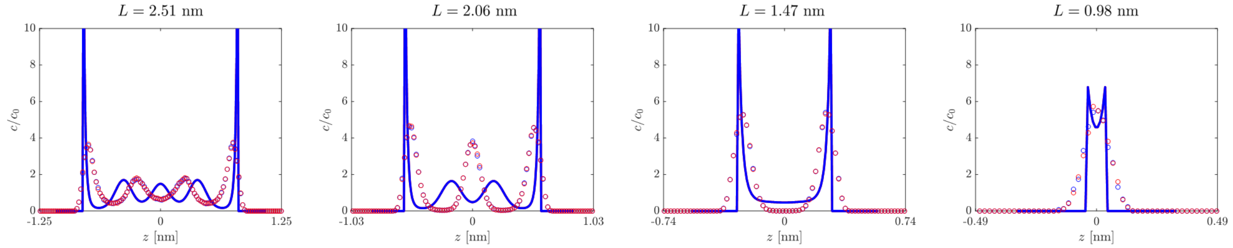

Figure S16: ($q_s = 0$ C/m^2, $d_i = 1.0\sigma_i$, Symmetric) Charge and ion density profiles in symmetric ILs between the charged plates. Markers, (○): simulations; Lines, (—): theory. Color coding: (—) - anions, (—) - cations.

2.5 Pressure Profile Comparisons, $d_i = 0.9\sigma_i$

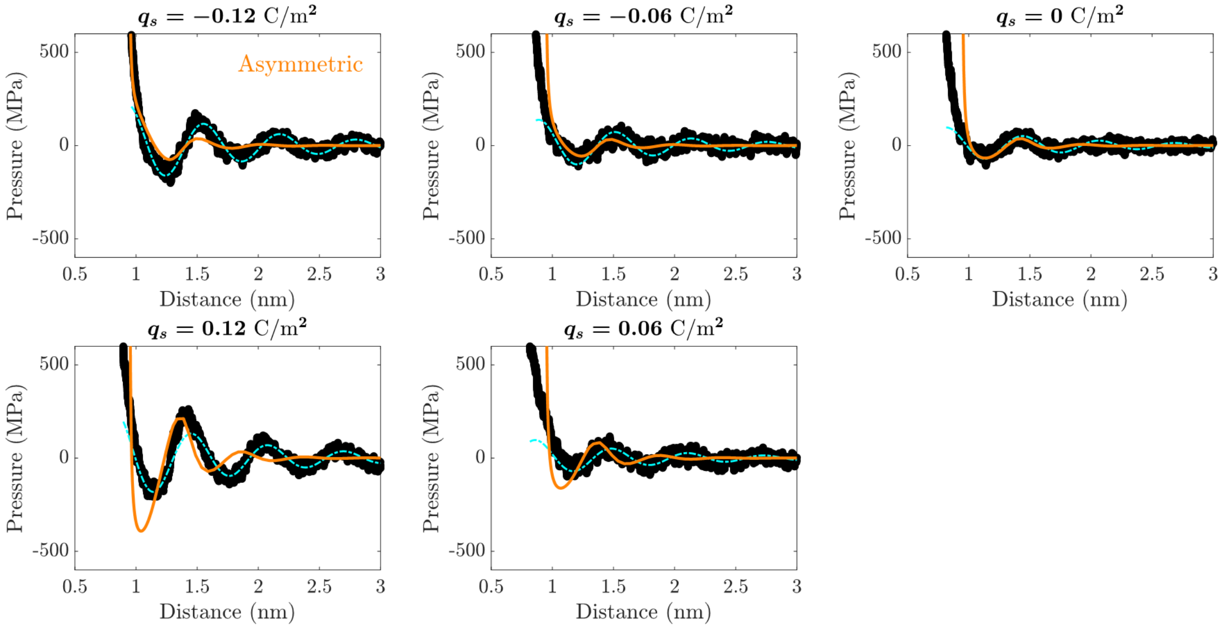

Figure S17: ($d_i = 0.9\sigma_i$, Asymmetric) Disjoining pressure profiles. The surface charge density is specified in the title of each plot. The black markers (○) are the MD simulation data points. The solid lines are the full, nonlinear integro-differential theory, where (—) corresponds to the asymmetric system. The other dashed and dash-dot lines are applications of the approximation in equation 14, where the parameters P_0 and z_0 are fit only to the first minimum. Here, the (- - -) lines correspond to the analytical expressions for κ in equations 28 and 29.

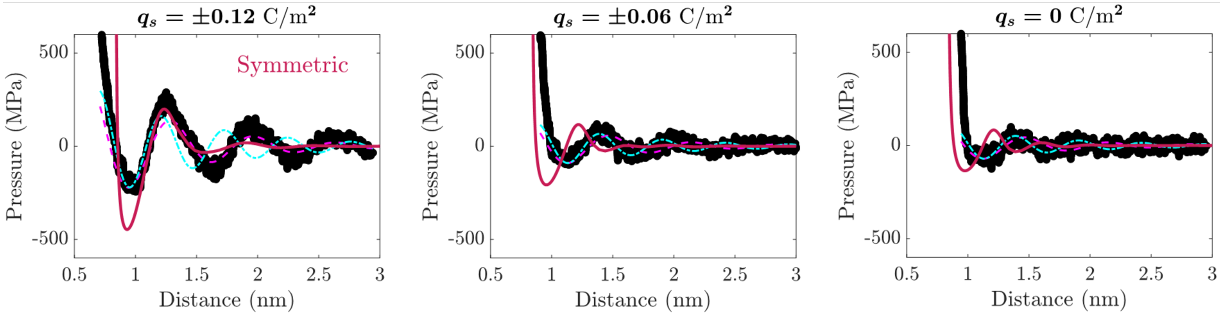

Figure S18: ($d_i = 0.9\sigma_i$, Symmetric) Disjoining pressure profiles. The surface charge density is specified in the title of each plot. The black markers (○) are the MD simulation data points. The solid lines are the full, nonlinear integro-differential theory, where (—) corresponds to the symmetric system. The other dashed and dash-dot lines are applications of the approximation in equation 14, where the parameters P_0 and z_0 are fit only to the first minimum. Here, the (- - -) lines correspond to the analytical expressions for κ in equations 28 and 29, while the (– – –) lines correspond to the definitions in equations 30 and 31.

2.6 Pressure Profile Comparisons, $d_i = 1.0\sigma_i$

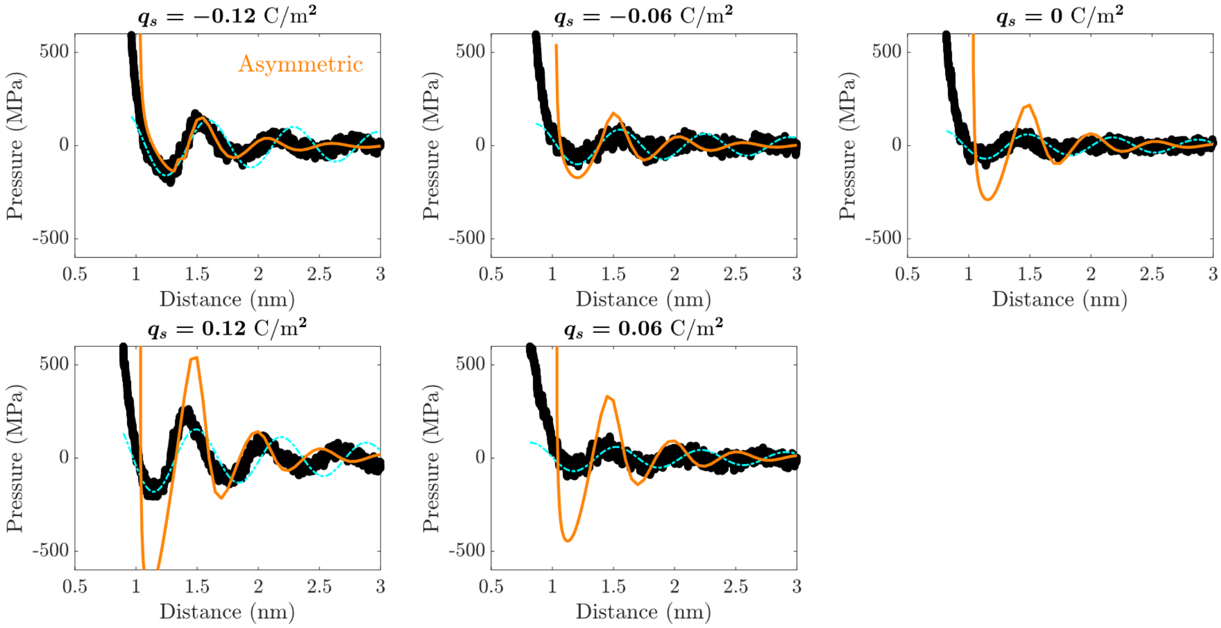

Figure S19: ($d_i = 1.0\sigma_i$, Asymmetric) Disjoining pressure profiles. The surface charge density is specified in the title of each plot. The black markers (○) are the MD simulation data points. The solid lines are the full, nonlinear integro-differential theory, where (—) corresponds to the asymmetric system. The other dashed and dash-dot lines are applications of the approximation in equation 14, where the parameters P_0 and z_0 are fit only to the first minimum. Here, the (- - -) lines correspond to the analytical expressions for κ in equations 28 and 29.

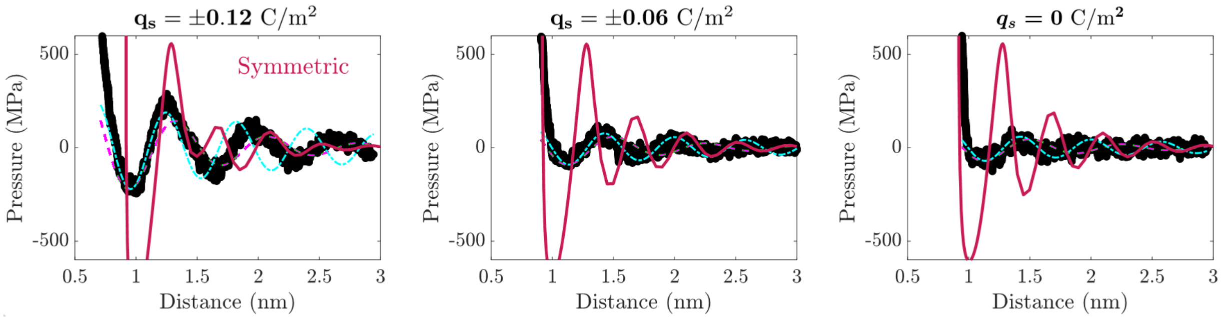

Figure S20: ($d_i = 1.0\sigma_i$, Symmetric) Disjoining pressure profiles. The surface charge density is specified in the title of each plot. The black markers (∘) are the MD simulation data points. The solid lines are the full, nonlinear integro-differential theory, where (—) corresponds to the symmetric system. The other dashed and dash-dot lines are applications of the approximation in equation 14, where the parameters P_0 and z_0 are fit only to the first minimum. Here, the (- - -) lines correspond to the analytical expressions for κ in equations 28 and 29, while the (– – –) lines correspond to the definitions in equations 30 and 31.

References

(1) Cámara, L. G.; Bresme, F. Molecular dynamics simulations of crystallization under confinement at triple point conditions. *The Journal of chemical physics* **2003**, *119*, 2792–2800.

(2) Gao, J.; Luedtke, W.; Landman, U. Structure and solvation forces in confined films: Linear and branched alkanes. *The Journal of chemical physics* **1997**, *106*, 4309–4318.

(3) Gao, J.; Luedtke, W. D.; Landman, U. Friction control in thin-film lubrication. *The Journal of Physical Chemistry B* **1998**, *102*, 5033–5037.

(4) Fedorov, M. V.; Kornyshev, A. A. Ionic liquid near a charged wall: Structure and capacitance of electrical double layer. *The Journal of Physical Chemistry B* **2008**, *112*, 11868–11872.

(5) Fajardo, O.; Bresme, F.; Kornyshev, A.; Urbakh, M. Electrotunable lubricity with ionic liquid nanoscale films. *Scientific reports* **2015**, *5*, 1–7.

(6) Fedorov, M. V.; Kornyshev, A. A. Ionic Liquids at Electrified Interfaces. *Chem. Rev.* **2014**, *114*, 2978–3036.

(7) Merlet, C.; Rotenberg, B.; Madden, P. A.; Taberna, P.-L.; Simon, P.; Gogotsi, Y.; Salanne, M. On the molecular origin of supercapacitance in nanoporous carbon electrodes. *Nature materials* **2012**, *11*, 306–310.

(8) Thompson, P. A.; Robbins, M. O. Origin of stick-slip motion in boundary lubrication. *Science* **1990**, *250*, 792–794.

(9) Erlandsson, R.; Hadziioannou, G.; Mate, C. M.; McClelland, G.; Chiang, S. Atomic scale friction between the muscovite mica cleavage plane and a tungsten tip. *The Journal of chemical physics* **1988**, *89*, 5190–5193.

(10) Smith, A. M.; Perkin, S., et al. Switching the structural force in ionic liquid-solvent mixtures by varying composition. *Physical review letters* **2017**, *118*, 096002.

(11) Pivnic, K.; Bresme, F.; Kornyshev, A. A.; Urbakh, M. Structural forces in mixtures of ionic liquids with organic solvents. *Langmuir* **2019**, *35*, 15410–15420.

(12) Capozza, R.; Vanossi, A.; Benassi, A.; Tosatti, E. Squeezout phenomena and boundary layer formation of a model ionic liquid under confinement and charging. *The Journal of chemical physics* **2015**, *142*, 064707.

(13) Horn, R. G.; Israelachvili, J. N. Direct measurement of structural forces between two surfaces in a nonpolar liquid. *The Journal of Chemical Physics* **1981**, *75*, 1400–1411.

(14) Berendsen, H. J.; van der Spoel, D.; van Drunen, R. GROMACS: a message-passing parallel molecular dynamics implementation. *Computer physics communications* **1995**, *91*, 43–56.

(15) Bussi, G.; Donadio, D.; Parrinello, M. Canonical sampling through velocity rescaling. *The Journal of chemical physics* **2007**, *126*, 014101.

(16) Berendsen, H. J.; Postma, J. v.; van Gunsteren, W. F.; DiNola, A.; Haak, J. R. Molecular dynamics with coupling to an external bath. *The Journal of chemical physics* **1984**, *81*, 3684–3690.
